# A Wearable Soft Robotic Exoskeleton for Hip Flexion Rehabilitation

**DOI:** 10.3389/frobt.2022.835237

**Published:** 2022-04-28

**Authors:** Tiana M. Miller-Jackson, Rainier F. Natividad, Daniel Yuan Lee Lim, Luis Hernandez-Barraza, Jonathan W. Ambrose, Raye Chen-Hua Yeow

**Affiliations:** ^1^ Evolution Innovation Lab, Advanced Robotics Centre, National University of Singapore, Singapore; ^2^ Department of Biomedical Engineering, National University of Singapore, Singapore

**Keywords:** soft robot, exoskeleton, soft pneumatic actuator (SPA), pneumatic rotary actuator, soft exosuit, lower limb rehabilitation, frugal innovation, rehabilitation robot.

## Abstract

Leg motion is essential to everyday tasks, yet many face a daily struggle due to leg motion impairment. Traditional robotic solutions for lower limb rehabilitation have arisen, but they may bare some limitations due to their cost. Soft robotics utilizes soft, pliable materials which may afford a less costly robotic solution. This work presents a soft-pneumatic-actuator-driven exoskeleton for hip flexion rehabilitation. An array of soft pneumatic rotary actuators is used for torque generation. An analytical model of the actuators is validated and used to determine actuator parameters for the target application of hip flexion. The performance of the assembly is assessed, and it is found capable of the target torque for hip flexion, 19.8 Nm at 30°, requiring 86 kPa to reach that torque output. The assembly exhibits a maximum torque of 31 Nm under the conditions tested. The full exoskeleton assembly is then assessed with healthy human subjects as they perform a set of lower limb motions. For one motion, the Leg Raise, a muscle signal reduction of 43.5% is observed during device assistance, as compared to not wearing the device. This reduction in muscle effort indicates that the device is effective in providing hip flexion assistance and suggests that pneumatic-rotary-actuator-driven exoskeletons are a viable solution to realize more accessible options for those who suffer from lower limb immobility.

## 1 Introduction

Leg motion is something many take for granted; however, the faculty for leg motion can be impaired. This work is focused specifically on those who suffer from hip flexor weakness. The causes for hip flexor weakness can include hip injury, stroke, and degeneration due to aging or disease. In the United States, over 300,000 people 65 and older are hospitalized for hip fractures yearly (Centers for Disease Control and Prevention 2016), and stroke reduces mobility in more than 50% of stroke survivors age 65 and over (Centers for Disease Control and Prevention 2021).

Robotic exoskeletons, devices worn on the body for rehabilitation, have been developed. Lokomat (Krishnan et al., 2013) and LOPES (Veneman et al., 2007) are examples of such devices which have been found to be highly effective for lower limb rehabilitation. HAL (Sczesny-Kaiser et al., 2019) and ReWalk (Kwon et al., 2020) are examples of similar but more portable lower limb exoskeletons. Given the impressive utility of lower limb rehabilitation devices, they are not as ubiquitous as would be expected. A possible factor could be cost. According to the most recent data from The World Bank, the average health expenditure per capita for one entire year for “Low and middle income” countries was only around 271 USD, and this group makes up the majority of the world population (The World Bank 2022). Thus, it would be valuable to realize a device with rehabilitative capabilities yet made from materials which are low-cost.

Opportunely, there is an emerging subset of robotics known as soft robotics. Soft robotic actuators are made from flexible materials such as silicon, textiles, flexible plastics, or cables (Ilievski et al., 2011; Kim et al., 2013; Rus and Tolley 2015; Wang et al., 2015). They are highly compliant and can thus offer a safer human-robot interaction. (Cianchetti et al., 2014; Low et al., 2014; Yap et al., 2015). They tend to be lighter than traditional robotic actuators, which affords surprising force-to-weight ratios (Yap et al., 2016; Khin et al., 2017). Furthermore, due to their fabrication materials, they can be manufactured at low costs (Chou and Hannaford 1996; Brown et al., 2010; Hiller and Lipson 2012; Wang et al., 2015).

There are various modes of soft robotic actuation such as cable-pulley systems (Asbeck et al., 2015b; In et al., 2015), dielectric elastomers (Carpi et al., 2010; Kocis and Knoflicek 2017), and pneumatics (Low et al., 2016; Low et al., 2017; Sun et al., 2017; Natividad et al., 2020). Cable-pulley systems are one of the more explored means of actuation for lower limb exoskeletons (Ding et al., 2014; Awad et al., 2017; Ding et al., 2017). Xiloyannis et al. point out several advantages and limitations of this method (Xiloyannis et al., 2021). For advantages, they are driven by electric motors, which have well-studied control mechanisms. They also tend to use Bowden cable sheaths which can easily be routed across the body. As for limitations, they apply high shear forces to wearers, and they offer low mechanical efficiency. Cable-pulley systems can be employed in complement with other soft actuators, such as pneumatics, as exhibited by (Meng et al., 2020). Pneumatic actuation tends to be relatively slow, since it depends on the pressurization of soft chambers which is often limited by air flowrate, but cable-pulley systems can actuate quickly. Pneumatic actuation has its own advantages. Compared to other types of soft actuators such as dielectric elastomers and shape memory allows, soft pneumatic actuators (SPAs) can be made more easily and at lower cost and can be safter to operate and provide more flexibility (Sun and Chen 2014). Pneumatic actuation is the mechanism chosen for this work.

Some SPAs have been successfully employed in lower limb exoskeletons. A contracting type of SPAs, pneumatic actuator muscles (PAMs) have been used in lower limb exoskeletons to assist ankle and/or knee motion (Sawicki and Ferris 2009; Park et al., 2014a; Park et al., 2014b; Li et al., 2019) and some have been proposed for walking assistance (Diteesawat et al., 2018). The CPAM (curl pneumatic artificial muscle) is a variation of the PAM which curls as it contracts. Wang et al. designed a CPAM-driven lower limb device which assists hip, knee, and ankle joints (Wang et al., 2020).

While useful, PAMs may be limited by stroke length, since their motion depends on contraction. A larger range of motion may be afforded by PRAs (pneumatic rotary actuators) (Sridar et al., 2017; Chung et al., 2018; Tiana M. Miller-Jackson et al., 2019; Veale et al., 2019; Fang et al., 2020; Park et al., 2020; Sridar et al., 2020; Veale et al., 2021), a type of SPA which produces rotary motion. J. Chung et al. presented the PRA-driven Exoboot for ankle motion assistance and reported a maximum torque of 39 Nm at a supply pressure of 483kPa and an ankle angle of 60° (Chung et al., 2018). A. J. Veale et al. presented the pleated pneumatic interference actuator for knee extension assistance for sit-to-stand motion, which can provide an impressive 324 Nm of torque at 320 kPa and a knee angle of 82° of flexion (Veale et al., 2021).

Despite these promising results of PRA-driven lower-limb exoskeletons, not many groups have reported the efficacy of their device in assisting actual human subjects. Of the few examples, S. Sridar et al. presented a PRA-driven device for knee extension assistance and reported a 7% reduction in muscle effort for walking (Sridar et al., 2017). J. Fang et al. also presented a knee extension device, reporting a maximum reduction of 64.21% muscle signal for the rectus femoris in the half squat motion (Fang et al., 2020). Yang et al. presented a hip abduction device and reported a maximum reduction of 43% for gluteus maximus (Yang et al., 2020).

One reason for lack of human testing is the challenge of actuating SPAs at a speed commensurate with normal human motion. J. Park et al. have proposed a design in which some sections remain inflated at all times (inactive sections) so that only certain sections must be inflated for actuation (active sections) (Park et al., 2020). This reduces actuation volume resulting in faster inflation. S. Sridar et al. have chosen, rather, to adopt a hybrid design of both soft and rigid components in (Sridar et al., 2020). Rigid lateral extensions attached to a soft central pneumatic section allow this hybrid actuator to meet the same torque output per pressure of an equally-sized fully soft actuator, yet with a 4.5 times faster rise time.

Given the positive results described, PRAs may be viable to produce effective lower limb exoskeletons. However, as stated, there are not many publications which report the efficacy of PRA-powered exoskeletons in assisting human subjects. Furthermore, nearly no devices are targeted toward hip assistance, though many are targeted toward knee and ankle assistance. Therefore, it is the aim of this work to present a PRA-driven exoskeleton for hip flexion assistance and demonstrate its efficacy in providing assistance with real human subjects.

To avoid wearer injury, use of the device should follow certain requirements. Firstly, the device should only be used by wearers who are able to support their own bodyweight in a standing position. Secondly, the device should have restrictive measures to limit its range of motion so as not to injure the wearer. For example, in this work, the device range of motion was limited to approximately that of a normal human hip range of motion during walking, namely 30° flexion and 15° extension. Additionally, torque output of the device should be restricted to prevent discomfort or injury to the wearer. In this work, torque output is restricted through controlling the inlet air flow to the pneumatic actuator.

## 2 Materials and Methods

### 2.1 The Soft Wearable Assistant for Gait

**FIGURE 1:**
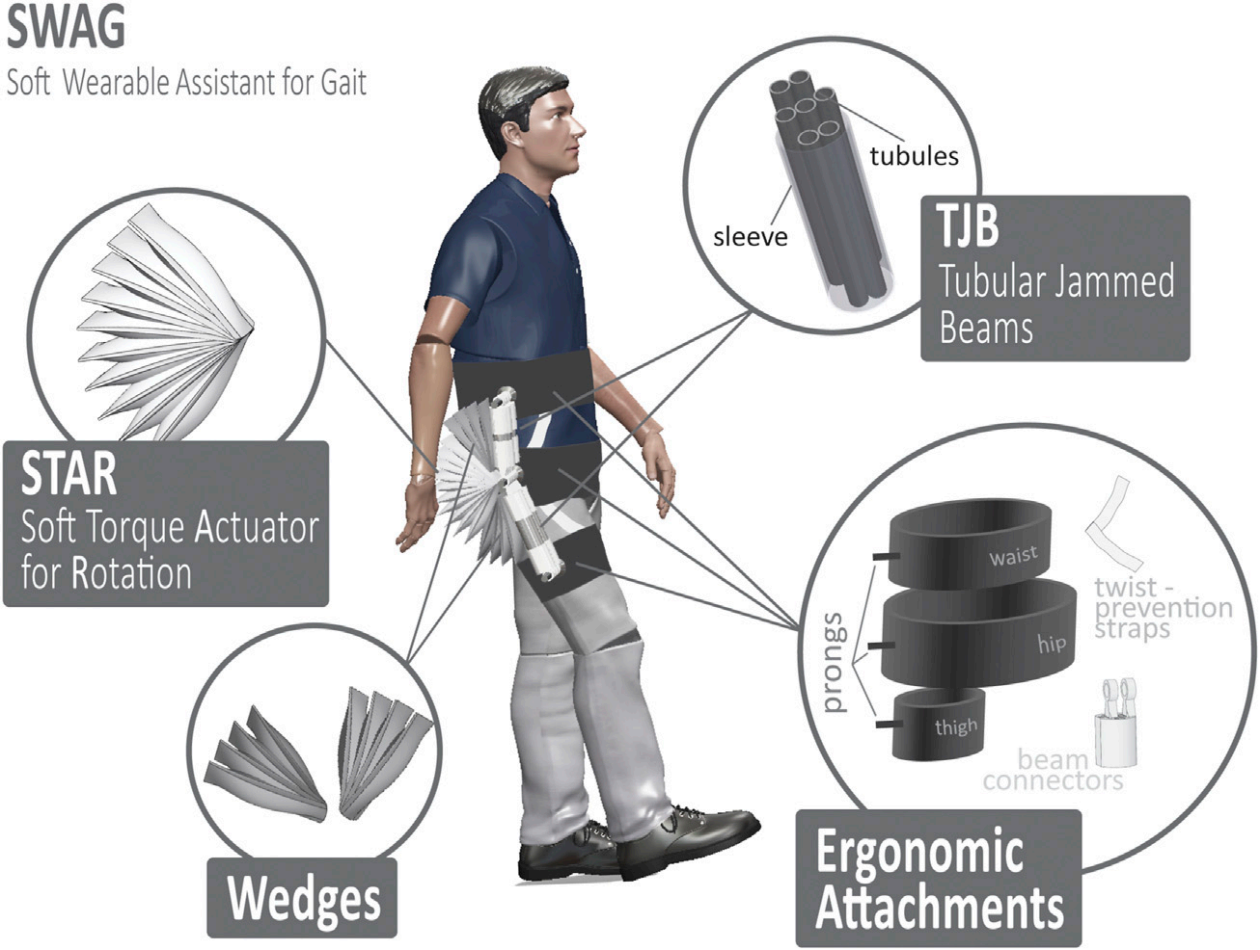
Overview of soft wearable assistant for gait (SWAG).

The Soft Wearable Assistant for Gait (SWAG) is a wearable exoskeleton which assists with hip flexion. The device uses pneumatic rotary actuators (PRAs) to transmit torque to the leg in the direction of hip flexion. The SWAG is comprised of tubular jammed beams (TJBs) (Tiana Miller-Jackson et al., 2019), an array of PRAs, and strategic ergonomic attachments (Figure 1).

#### 2.1.1 Tubular Jammed Beams

In our previous work, we have introduced tubular jamming, a variable stiffening method for soft actuators which requires only positive pressure (Tiana Miller-Jackson et al., 2019). Tubular jammed beams (TJBs) are soft beams with variable stiffness. They are comprised of a manifold of inflatable tubules housed inside a retaining sleeve. When unpressurized, the TJBs are highly compliant. This feature is advantageous for use in the exoskeleton as it enables the device to be positioned to fit each user’s unique body shape. When pressurized, the TJBs become stiff and fixed in their preset position. Once stiffened they become load-bearing beams which transmit the assistive torque from the PRAs to the user’s leg for assistance. In this work, each TJB contained five tubules of diameter 50/π mm in sleeves of diameter 120/π mm. Two TJBs were used in each section of the device (Figure 1). The length for the top section was 11.5 mm and the length for the bottom section was 16.5 mm.

#### 2.1.2 Soft Torque Actuator for Rotation

The Soft Torque Actuator for Rotation (STAR, Figure 1) is comprised of multiple PRAs (Khin et al., 2017) based on those presented in our previous work (Tiana M. Miller-Jackson et al., 2019). The STAR is tethered to a central rotation point called a prong, which is part of the ergonomic attachments. Pressurization of these actuators causes spatial interference between them which generates an expansive force. Because the actuators are tethered to a common axis, this motion is translated into torque. This torque is then captured by the TJBs and transferred into the body of the user in the trajectory of hip flexion. Thus, the STAR is the driving actuation component of the device. The STAR has an adjustable safety tether which restricts rotation to a maximum angle. This feature keeps the device within a safe range of motion and prevents it from applying forces to the user in unwanted trajectories.

#### 2.1.3 Wedges

Since the SWAG aligns with the wearer’s back and the wearer’s thigh (see Figure 1), its actuation array must span the large angular range from the back to the thigh (180° for neutral standing position and even larger angular range for hip flexion position). Through observation of the mechanical behavior of a PRA (as in (Tiana M. Miller-Jackson et al., 2019)) it is evident that its torque output is maximized when its angular position is minimized, so minimal PRA angles are desirable. That is, a large angular range is required for the actuation array, yet, it is desirable to minimize the angular range of each individual PRA. Thus, the Wedges are introduced. These components are inactive sections of PRAs which remain in static angular position throughout device operation. This minimizes the angular positions required of the active PRAs in the STAR section, which enables the large torque output required, even at maximum device angular positions. Furthermore, the static Wedges enable a smaller actuation motion for the STAR section, which leads to faster actuation speed.

#### 2.1.4 Ergonomic Attachments

The SWAG is attached to the user in three locations, the torso, the hip joint, and the thigh. The torso attachment wraps around the user’s back to provide a strong base for the device to push against to lift the leg. This follows physiological architecture wherein the primary muscles responsible for lifting the leg are anchored to the back. The hip attachment aligns the axis of the device with the axis of the user’s hip joint. The flexibility of the device allows it to mold to the body shape of each user. This alignment is important because misalignment of device and user hip axes could lead to discomfort and inefficiency as forces will not be directed appropriately. The hip attachment anchors the STAR, which drives the exoskeleton to move. At the distal end of the device is the thigh attachment. Here, the torque from the STAR is transmitted through the TJBs to the leg and aids in hip flexion.

For all three attachments, the surface area which contacts the user was made maximally broad to distribute the force across as large an area as possible. This decreases the normal pressure experienced by the user, which affords a higher level of comfort (Asbeck et al., 2013; Asbeck et al., 2015b; Karavas et al., 2017). Furthermore, care was taken to route the attachments over bony segments wherever possible. When the skin is compressed against bony areas of the body, its displacement is notably lower than when compressed against other areas of muscle or fat (Asbeck et al., 2013). Displacement of the attachments can lead to user discomfort, device misalignment, and weakened efficacy. Thus, adjustable straps were incorporated to better maintain attachment positions on the user’s body. The twist-prevention straps are crucial to efficient operation of the device, as, without them, the actuation energy of the STAR would cause waist and thigh attachments to rotate around the user’s body rather than providing hip flexion assistance. Therefore, the straps are strategically positioned antagonistically to oppose this unwanted rotation movement so that force is pointed in the forward direction at the waist and thigh, as is desired.

#### 2.1.5 Control and Bandwidth

The SWAG device is controlled pneumatically using an electropneumatic regulator (ITV 2030; SMC Corporation, Tokyo, Japan). The device operation speed is controlled by adjusting the regulator on and off timing. Assistive torque is then delivered at fixed intervals based on the pre-selected, target motion speed. Since the device actuation system is soft and compliant, it will move with the wearer to any angular position within range. Thus, the angular position bandwidth of the device is governed by the angular position bandwidth of the wearer. The pressure bandwidth of the device is dependent on its angular position, as the increase of angular position decreases pressure (discussed further in sections 2), making definition of device bandwidth difficult to directly define. Based on pilot testing, the minimum cycle time for comfortable walking is 1.5 s. Thus device bandwidth is defined as 0.67 Hz.

### 2.2 Design Parameters

#### 2.2.1 Device Parameters

Walking is one of the most foundational tasks of everyday living which involves hip flexion motion, thus it is chosen to guide the design goals of the device. In walking, the hip reaches approximately 30° of flexion (Ramakrishnan and Kadaba 1991), thus, 30° is taken as the nominal target hip flexion angle for the device to assist the user to reach. Additionally, the hip reaches approximately 15° of extension during walking (Parvataneni et al., 2009). Therefore, SWAG range of motion should be designed such that it allows the user to reach that extension angle.

Based on the biomechanics data set forth by winter in (Winter 2009), and assuming a nominal body mass of 62 kg (Walpole et al., 2012), the nominal static torque required to hold the leg at 30° hip flexion is 19.8 Nm. In order to determine necessary dimensions for the pneumatic rotary actuators to achieve this target torque, a physical model was developed based on the model presented by Felt (Felt 2019).

#### 2.2.2 Physical Model

**FIGURE 2:**
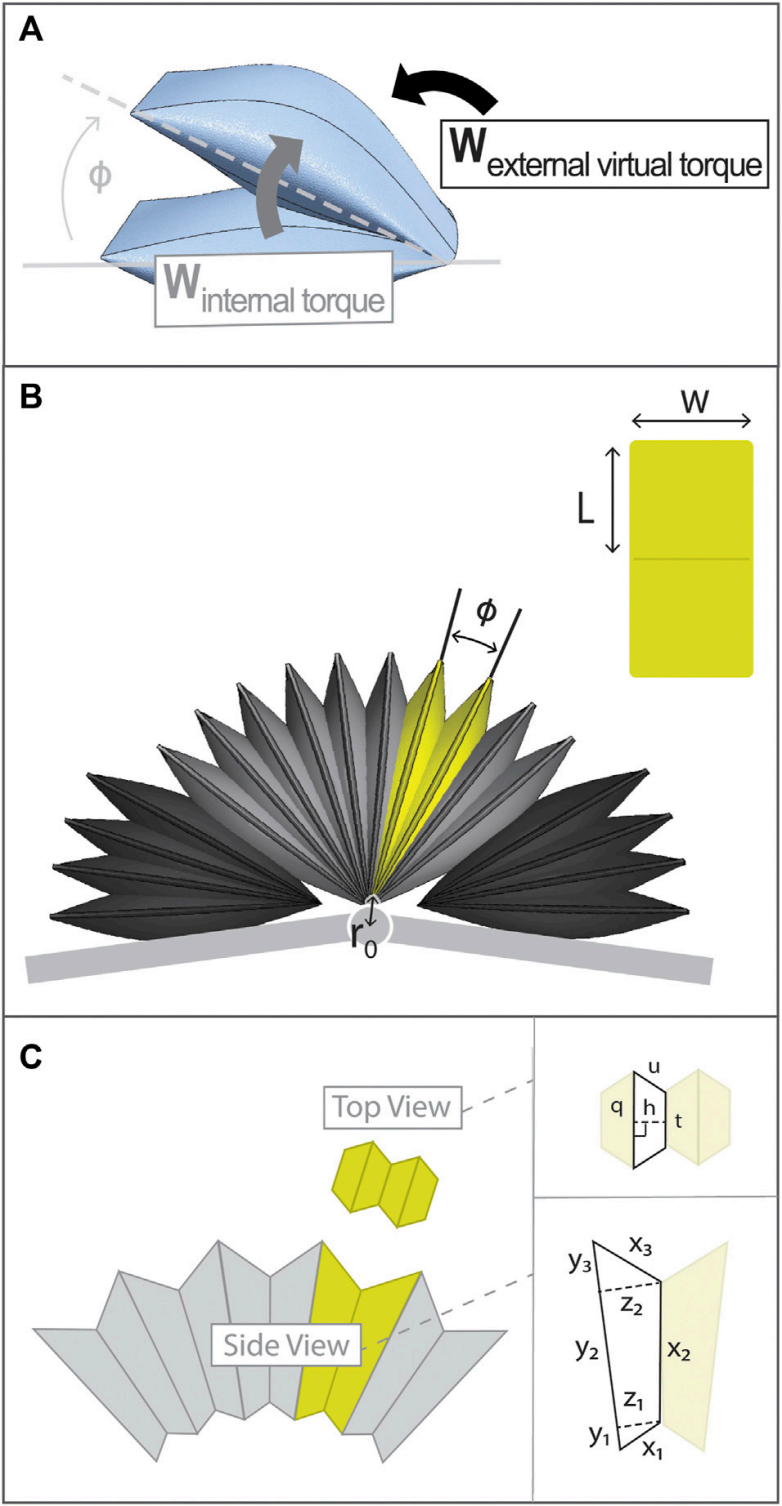
Details of Torque Model and PRA architecture. **(A)** Diagram of internal work and external virtual work of PRA which are set in equilibrium to determine torque output of PRA. **(B)** Entire actuation assembly with a single PRA highlighted in yellow and a view of PRA laid flat to show its dimension labels. **(C)** PRA volume is approximated using geometric shapes, with dimensions labels as shown.

Due to the laws of conservation, for a system in static equilibrium, the sum of work is zero. Therefore, taking the fabric of the PRAs to be inextensible, it holds that the sum of the work done by an external virtual torque (W_T_) and the internal work done by the change in volume of the structure (W_i_) should be zero (Figure 2A):

$$\delta W_{T}\;+\delta W_{i}\;=0$$ (1)

The change in external work (δW_T_) and the change in internal work (δW_i_) can be expressed as follows:

$$\begin{array}{l} \delta W_{T}\;=T\cdot \delta \varphi \\ \delta W_{i}\;=\;P_{i}\;\cdot \mathrm{\delta V} \end{array}$$ (2)

where T is torque, δφ is change in angle, P_i_ is internal pressure, and δV is change in volume. Substituting eq. 2 into eq. 1, an expression for torque generated by the actuator can be found, where actuator torque (*τ*) is equal and opposite to the external virtual torque (T).

$$\mathrm{\tau =-}\mathrm{Τ=}P_{i}\cdot \mathrm{\delta V}/\mathrm{\delta \varphi =}P_{i}\cdot \mathrm{dV}/\mathrm{d\varphi}$$ (3)

The architecture of a PRA is presented in Figure 2B. The two sides of the PRA are denoted as arms and the point of the fold between them is the vertex. The characteristic dimensions of a PRA are shown, being namely angular position (*φ*), width (w), and length, where L is the length of one arm. As mentioned, each PRA is tethered to a common axis point, the prong. The distance from each PRA vertex to this axis point is denoted as r_0_.

First, the shape of each PRA is approximated using standard geometric shapes, as shown in Figure 2C, where: y_1_, y_2_, and y_3_ make up the length of the outer surface of one PRA arm; x_1_, x_2_, and x_3_ make up the length of the inner surface of one PRA arm; and z_1_ and z_2_ are the widths, perpendicular to the y surface, at the points shown. Segment x_2_ is the length of the contact area between the two arms of the PRA; this contact area produces torque.

Widths z_1_ and z_2_ can be expressed in terms of r_0_, y_1_, and y_2_:

$$\begin{array}{l} z_{1}\;=\;\left(r_{0}\;+\;y_{1}\right)\tan\left(\varphi /2\right)\\ z_{2}\;=\;\left(r_{0}\;+\;y_{1}\;+\;y_{2}\right)\tan\left(\varphi /2\right)\; \end{array}$$ (4)

The length of the x surface is equated to PRA arm length (L), in which lengths x_1_, x_2_, and x_3_ can be expressed in terms of y and z parameters. This allows for y_3_ to be expressed in terms of y_1_ and y_2_:

$$\begin{array}{l} \text{L}={\text{x}}_{1}+{\text{x}}_{2}+{\text{x}}_{3}\\ \text{L}=\sqrt{y_{1}^{2}+\;z_{1}^{2}}\;\;+\;\;\sqrt{y_{2}^{2}\;+\;{\left(z_{2}-z_{1}\right)}^{2}\;}\;\;+\;\;\;\sqrt{y_{3}^{2}+\;z_{2}^{2}}\\ y_{3}=\sqrt{{\left(L-\sqrt{y_{1}^{2}+\;z_{1}^{2}}-\sqrt{y_{2}^{2}\;+\;{\left(z_{2}-z_{1}\right)}^{2}\;}\right)}^{2}-z_{2}^{2}} \end{array}$$ (5)

The top-view cross section of the PRA can be approximated as four trapezoids (Figure 2C), where h is trapezoid height, q is the length of the larger trapezoid base, t is the length of the smaller trapezoid base, and u is the length of the leg. The area of the trapezoid can then be found as follows:

$$A=\frac{q+t}{2}h$$ (6)

The sum of the trapezoid legs (u) and smaller base (t) must be equal to the width of the PRA (w). Thus, the larger trapezoid base (q) can be expressed in terms of h:

$$\begin{array}{l} u=\frac{1}{2}\left(w-t\right)\\ h=\frac{1}{2}\sqrt{{\left(w-t\right)}^{2}-{\left(q-t\right)}^{2}}\\ q=\sqrt{{\left(w-t\right)}^{2}-4h^{2}}+t \end{array}$$ (7)

The trapezoid height, h, varies along the length of the PRA defined by the width of the side-view cross section parameters as follows:

$$\mathrm{h=}\{\begin{array}{c} \frac{z_{1}}{2}\frac{\left(y-r_{0}\right)}{y_{1}},\;\;\;\;\;\;\;\;\;\;\;\;\;\;\;\;\;\;\;\;\;\;\;\;\;\;\;\;\;\;\;\;\;\;\;\;\;\;\;r_{0}\leq y\leq r_{0}+\;y_{1}\\ \frac{y\;\text{tan}\left(\phi /2\right)}{2}\;,\;\;\;\;\;\;\;\;\;\;\;\;\;\;\;\;\;\;\;\;\;r_{0}+\;y_{1}\leq y\leq r_{0}+y_{1}+y_{2}\\ \frac{z_{2}}{2}\left(1-\frac{y-r_{0}-y_{1}-y_{2}}{y_{3}}\right),\;\;r_{0}+\;y_{1}+y_{2}\leq y\;\leq r_{0}+\;y_{1}+y_{2}+y_{3} \end{array}$$ (8)

Since all parameters can be expressed in terms of y_1_ and y_2_, it is left only to find the values of these two variables. It is given that the PRA fabric always moves to the resting shape which maximizes volume to minimizes strain energy. Thus, y_1_ and y_2_ can be found by determining the values of each which maximize PRA volume, V. Recall that A is the area of one trapezoid of the top-view cross section, therefore four trapezoids make up the total cross-sectional area of the PRA in that plane.

$$V=\;4\cdot \int_{r_{0}}^{r_{0}+\;y_{1}+y_{2}+y_{3}}A\left(y\right)\;\mathrm{dy}$$ (9)

Where, combining (6) and (7)

$$A\left(y\right)=\frac{\sqrt{{\left(w-t\right)}^{2}-4h^{2}}+2t}{2}h$$ (10)

The values of h and t which maximize A(y) were determined by solving the partial derivatives of A(y) with respect to each.

$$\begin{array}{l} h_{\max}=\frac{\sqrt{3}}{6}w\\ t_{\max}=w-\frac{4\sqrt{3}}{3}h\\ A_{\max}\left(y\right)=w\;h\left(y\right)-\sqrt{3}\;{\left[h\left(y\right)\right]}^{2} \end{array}$$ (11)

Given this expression, parameters y_1_ and y_2_ which maximize volume can be found. Consequently, the volume at each angle *φ* can be determined and, using eq. 3, the torque generated by the PRA at each angle *φ* can be found. To validate this model, it was applied to a set of PRAs of differing dimensions as presented in previous work and compared with measured torque data (Tiana M. Miller-Jackson et al., 2019). An angle range of 10°–30° was assessed, since it is expected that only a small PRA angular movement will be needed. Subsequently, results were used to select PRA parameters to generate adequate torque required for the chosen application of hip flexion assistance.

### 2.3 Performance Testing

#### 2.3.1 Torque Test

**FIGURE 3:**
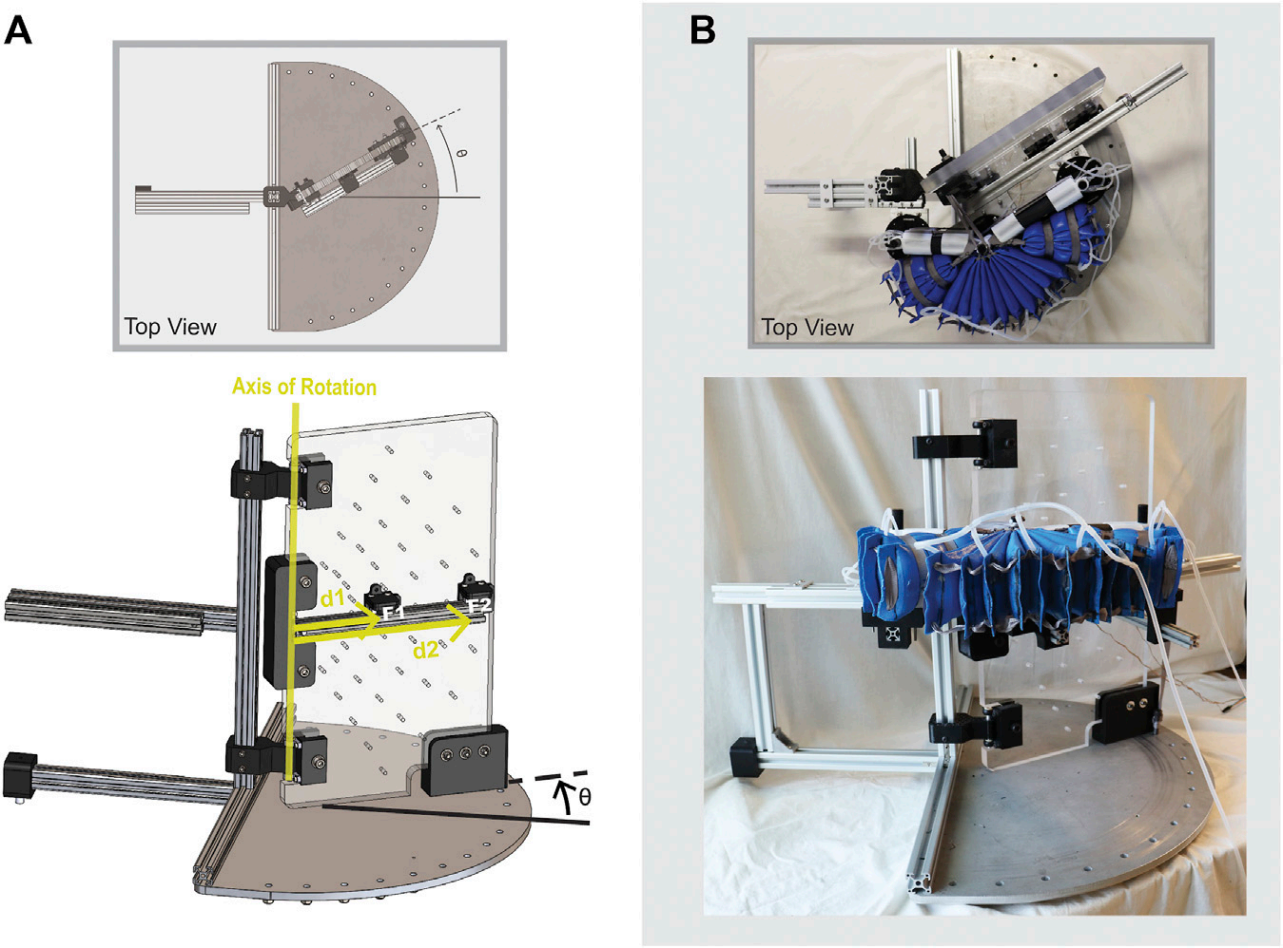
Configuration to measure torque output of SWAG actuation system using custom torque measurement apparatus. Adjustable arm is rotated to set angle *θ*.

Torque output is a critical performance metric of the device since it determines how much assistance the device is able to provide to the user. Due to the geometry of the device as laid out in the model section, the torque output varies with PRA angle. Because of the flexible nature of the device and its attachment points, it is anticipated that there will be some torque transmission loss from the STAR to the user. Therefore, the fully assembled actuation system was assessed, including the STAR, Wedges, and TJBs. An adequately-sized torque measurement apparatus was fitted with adaptors to connect the actuation system (Figure 3). The apparatus has one stationary arm and one adjustable arm so that the position of the device can be constrained at custom discrete angles. Apparatus angle, *θ*, is measured as shown in Figure 3A. This reference frame is chosen so that *θ* corresponds with the hip flexion angle of a human user. Force is detected by load cell sensors at the adjustable arm (F_1_ and F_2_) and is converted to torque (*τ*_Total_) based on the distance from each sensor to the axis of rotation (d_1_ and d_2_, as shown in Figure 3A):

$$F_{1}\cdot d_{1}\;+\;F_{2}\cdot d_{2}\;=\;\tau_{\mathrm{Total}}$$ (12)

The adjustable arm was set at fixed angles from *θ* = 0 to *θ* = 30°, in increments of 10°. For each angular position, the pressure was varied from 20 to 100 kPa, in increments of 10 kPa, and the resulting torque output was recorded.

#### 2.3.2 Dynamic Pressure Test

**FIGURE 4:**
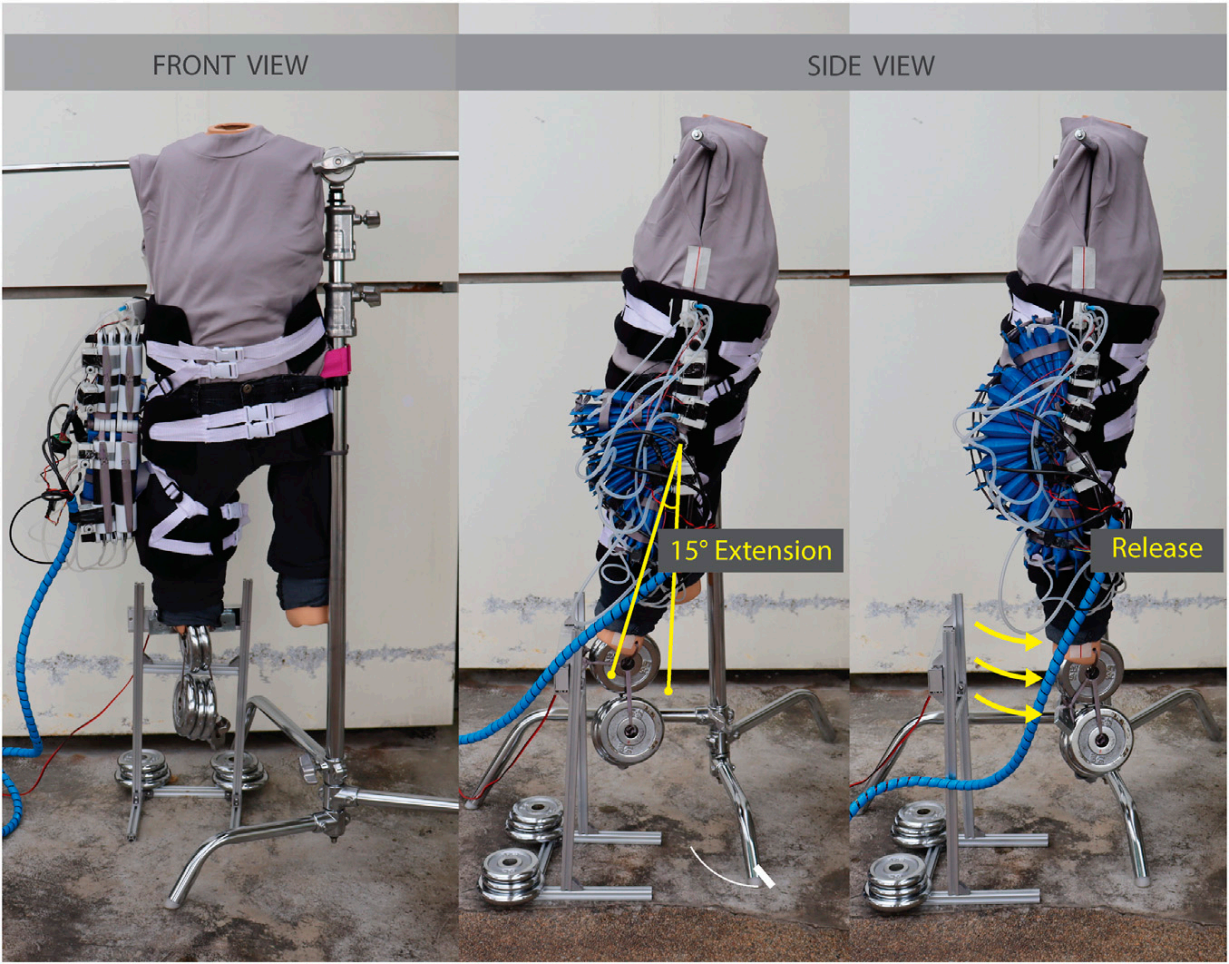
Configuration for dynamic pressure test.

Since the device is intended to be used primarily for dynamic assistance, it is important to assess the actuation system under dynamic conditions. The actuation system was joined with the ergonomic attachments to form the complete SWAG device, and a dynamic pressure tests was performed. The device was fitted on a mannequin with movable joints to simulate its functional use with a human wearer as closely as possible (shown in Figure 4). Weights were added to the leg of the mannequin to achieve a more realistic leg mass of 10 kg. In use with a human wearer, the actuators are activated when the hip of the wearer is in an extended position, at around 15° of extension. To simulate this, the leg of the mannequin was pulled back into a position of approximately 15° of hip extension, measured using a goniometer, and held in that position using an electromagnet. Power to the electromagnet was integrated with the pneumatic control system such that the start of actuator pressurization and electromagnet release were simultaneously triggered. The pressure was recorded and the dynamic pressurization of the device was assessed. The supply air pressure was set to 500 kPa and an electropneumatic solenoid valve (VDW350; SMC Corporation, Tokyo, Japan) was used to open and close the supply pressure. The supply pressure was open at the start of a trial and triggered to close when the actuator pressure reached its target pressure of 86 kPa, as detected by a pressure transducer (MPX5500DP; NXP Semiconductors, Eindhoven, Netherlands). The measurement was performed five times and the values were averaged.

#### 2.3.3 Human Subject Test

After torque output and dynamic pressure evaluations were completed, the device was assessed in use by healthy human subjects. The aim of the study was to determine if the device is able to successfully provide hip flexion assistance to the user. The study was approved by the National University of Singapore Institutional Review Board (Protocol Title: Soft Robotic Lower Limb Assistant Assessment; Reference Code: LH-19-019C; Latest Version Approval Date: 13-July-2021).

Setting and Participants: The study was conducted in a gait laboratory with a runway for walking. Study participants comprised 4 females and 6 males of age 23 to 32 (average 26 ± 3) years, weight 45 to 75 (average 58 ± 10) kg, and height 155 to 180 (average 166 ± 10) cm. Participant criteria included walking with a regular gait pattern and being able to walk at least 500 m without impairment (pain, limping, etc.). For further descriptions of participants, see Table 1.

**TABLE 1:** Details of study participants.

#	Gender	Age (years)	Weight (kg)	Height (cm)
S01	M	25	75	178
S02	M	27	70	180
S03	M	24	58	158
S04	F	28	45	155
S05	M	24	62	170
S06	M	26	62	176
S07	F	23	50	159
S08	F	25	52	160
S09	M	32	58	168
S10	F	24	51	156
Avg	--	26 ± 3	58 ± 10	166 ± 10

**FIGURE 5:**
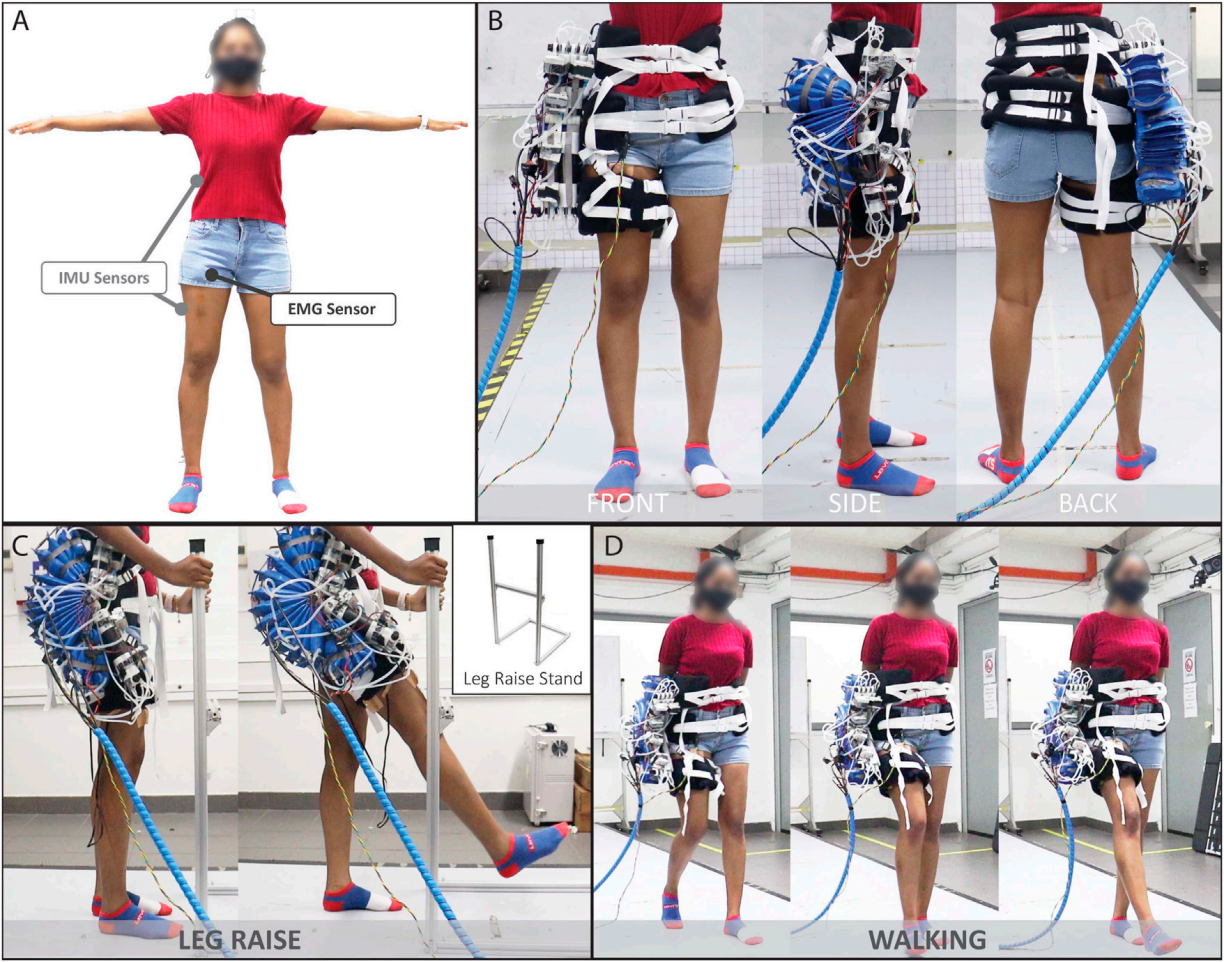
Human Subject Testing: **(A)** Approximate IMU sensor and EMG sensor placements, **(B)** Front, side and back views of the subject wearing the device **(C)** Example of subject performing the Leg Raise motion and **(D)** Walking motion, both in the assisted condition.

Testing Procedure: Two inertial measurement unit (IMU) sensors were attached to the subject’s skin, one at the torso and one on the right thigh. Approximate sensor position is shown in Figure 5A. Surface electromyography sensors (Trigno Wireless, Delsys Inc., US) were adhered to the rectus femoris of the right leg.

The subject was assessed under two conditions.

(1) Normal Baseline Measurement. The subject performed the motion without wearing the device.
(2) Assistance Measurement. The subject performed the motion while wearing the device, with the device powered on and providing assistance.

In each of the above conditions, the subject was asked to do a total of three motions: Leg Raise, Walk at 70 steps/min, and Walk at 80 steps/min. The Leg Raise motion was chosen as it is a core element of several other leg motions such as walking, running, kicking, and stairclimbing and can provide a baseline assessment of the efficacy of the device to assist in hip flexion for those more complex motions. Walking was chosen because it is one of the most essential motions to everyday life. The cadences of 70 steps/min and 80 steps/min were chosen to compare the efficacy of the device across operation speeds. These two specific cadences were selected because, in practice, subjects reported feeling unnaturally slow when walking slower than approximately 70 steps/min and the device actuation speed is currently limited to around 80 steps/min.

(1) Leg Raise. The subject was asked to stand on his or her left leg while holding the leg raise stand (shown in Figure 5C) or a nearby stool for support, as necessary. The subject was asked to lift his or her right leg to the front then lower back to neutral standing position, using the horizontal bar of the leg raise stand as a guide. Subjects were asked to avoid leaning but to stand up straight and look forward, to the extent possible, to improve hip flexion angle measurement accuracy with the IMU. For condition I, an audible metronome was played at 70 bpm and the subject was asked to synchronize his or her movements to its pace by lifting the leg in one beat and lowering the leg in two beats. The test administrator also provided verbal instruction to guide the subject. For condition II, the device was programmed to provide assistance at the same pace (70 bpm, with one beat to raise and two beats to lower), and the subject was asked to let the device move his or her leg at that pace. Figure 5C shows a subject performing the Leg Raise motion with assistance from the device.
(2) Walk at 70 steps/min. The subject was asked to walk at 70 steps/min. For condition I, an audible metronome was played at 70 steps/min and the subject was asked to synchronize his or her movements to its pace by taking one step per beat. For condition II, the device was programmed to provide assistance at a pace of 70 steps/min and the subject was asked to synchronize his or her movements by letting the device move the right leg and manually moving the left leg to sustain walking at that pace. Figure 5D shows a subject performing the walking motion with assistance from the device.
(3) Walk at 80 steps/min. The subject was asked to walk at 80 steps/min. The procedure was the same as that of the 70 steps/min walk, except at a cadence of 80 steps/min.

Data Analysis: Several trials were recorded for each subject. Afterward, trials were visually screened and erroneous trials were discarded. Individual motion cycles (leg raise cycle or gait cycle) were then segmented from successful trials based on flexion angle peaks and troughs. The cycles themselves were then sorted by motion criteria. For Leg Raise motion, cycles with a peak angle of 35 ± 10° and a starting angle of 0 ± 5° were assessed. For Walking motion, cycles within 5 steps/min of the respective target cadence (70 steps/min or 80 steps/min) were assessed. For assessed cycles, the EMG signal of rectus femoris was filtered with a 20 Hz high-pass filter (8th order Butterworth) to account for motion artifacts and muscle firing frequency. Next, a 48–52 Hz band stop filter (8th order Butterworth) was applied to account for power line noise. Outliers were then detected and removed, where outliers were defined as values more than 1.5 times the interquartile range beyond the upper or lower quartile. Subsequently, the data was rectified and the envelope was extracted using a 200-sample-wide moving average window. The data was then normalized by the maximum EMG signal obtained for the respective subject during the respective task under investigation (Halaki and Gi 2012; Whiteley et al., 2021). Finally mean and peak values were tabulated for the set of cycles for each subject for each motion (Leg Raise, Walk at 70 steps/min, and Walk at 80 steps/min) and under each condition (normal or assisted). The respective means of these resulting quantities (Mean Normalized EMG and Peak Normalized EMG) were then compared across the two conditions (Normal and Assisted) using a paired-sample *t*-test. Significance was determined as *p* < 0.05.

#### 2.3.4 Dynamic Torque Assessment

The dynamic torque output of the device was assessed while being worn by a human subject. The study was approved by the National University of Singapore Institutional Review Board (Protocol Title: Soft Robotic Lower Limb Assistant Assessment; Reference Code: LH-19-019C; Latest Version Approval Date: 13-July-2021). The subject was 75 kg in weight and 178 cm in height. Two IMU sensors were attached to the subject’s skin, one at the torso and one on the right thigh, as in Figure 5A. The subject performed Leg Raise motion, Walking at 70 steps/min, and Walking at 80 steps/min (8 trials of each motion), while his hip angle and the pressure of the actuator were recorded. The pressure and angle were then used to assess the estimated torque required by the subject and estimated torque output of the device during the motions. The torque required by the subject to perform the motion was estimated with nominal values and the following simple model:

$$\tau_{\mathrm{Req}}=\mathrm{mgL}\cdot \sin\left(\theta \right)$$ (13)

where m is the mass of the leg, g is the acceleration of gravity, L is the length from the hip joint to the leg center of mass, and *τ*_Req_ is the torque required to flex the hip to a given angle, *θ*. The torque output of the device was estimated using a second order surface fit based on the data derived from the torque test described in the previous section:

−1.736 + 0.4764 P – 0.2582θ −0 .0008806 P2 −0 .003949 P⋅θ+ 0.005378 θ2 (14)

where P is the recorded pressure of the actuator, and *θ* is the recorded hip angle of the wearer, which is taken to be the angle of the device. For this experiment, the supply pressure was set to 250 kPa and an electropneumatic regulator (ITV 2030; SMC Corporation, Tokyo, Japan) was used to control the supply pressure. For the Leg Raise motion and for Walking at 70 steps/min, the supply pressure was open for 0.86 s to achieve a motion speed of 70 cycles per minute. For Walking at 80 steps/min, the supply pressure was open for 0.75 s to achieve 80 cycles per minute.

## 3 Results

### 3.1 Physical Model

**FIGURE 6:**
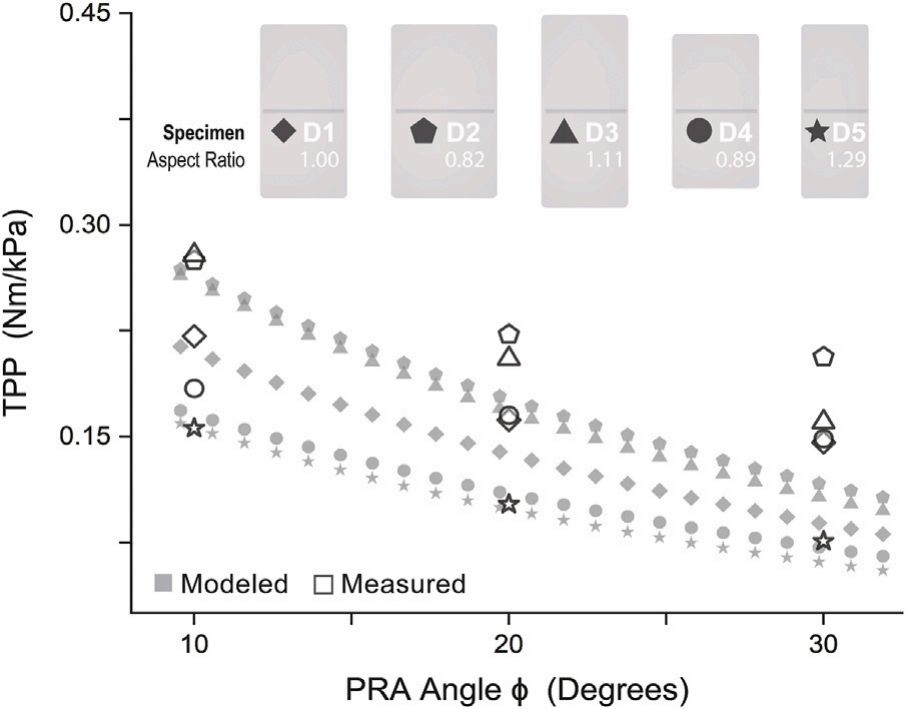
Model Validation: Physical model compared with measured results of torque per unit pressure varied by angle for PRAs of five different dimensions.

Our previous publication (Tiana M. Miller-Jackson et al., 2019) describes five PRAs of differing dimensions (Figure 6). The parameters of these PRAs were input into the aforementioned physical model to predict torque output per unit pressure (torque per pressure, or TPP) as a function of angular position. These results were then compared with measured TPP data reported in (Tiana M. Miller-Jackson et al., 2019).

As shown in Figure 6, the model is most accurate for D5 (mean model error 7.6%, max model error 19.2%) and least accurate for D4 (mean model error 31.5%, max model error 51.8%). Aside from specimen D2, an increase of model accuracy with increase of aspect ratio was observed, as shown in Table 2, where

Aspect Ratio = L/w (15)

$$\mathrm{Model error}=\;100\;\cdot \;\left|\mathrm{TP}P_{\mathrm{measured}}\;–\mathrm{TP}P_{\mathrm{model}}\right|/\mathrm{TP}P_{\mathrm{measured}}$$ (16)

**TABLE 2:** Aspect ratio and model error for varying PRA specimens.

Specimen	Length L (mm)	Width w (mm)	Aspect ratio (L/W)	Mean model error (%)
D2	90	110	0.82	22.3
D4	80	90	0.89	31.5
D1	90	90	1.00	19.2
D3	100	90	1.11	19.0
D5	90	70	1.29	7.6

Thus, it was concluded that this model is best suited for actuators with relatively large aspect ratios, It may be reasonably hypothesized that model accuracy increases for actuators with increasingly similar aspect ratio to those presented by Felt in (Felt 2019), the work from which the model presented here is derived. Leaving out specimen D2 and fitting a linear relation to aspect ratio and mean model error, the aspect ratio used by Felt (80/58 mm = 1.38) projects an error of nearly zero. Thus, it was determined to adopt this aspect ratio for the future design of PRAs in this work to optimize model accuracy.

In assessment of the efficacy of the physical model, the model curves accurately predict the trend of the TPP values in gradually decreasing as angle increases. (This decrease is due to the decrease in torque-generating spatial interference between actuator surfaces as angle increases, as mentioned previously.) However, it is evident that the model-predicted values of TPP decrease more rapidly than the measured values. Furthermore, all model-predicted values are underestimates across all dimensional variations of PRAs tested. While this does point to a shortcoming of the model for control purposes, this does not negate its use for design, and may prove to be a value, as actual torque produced will be satisfactory and likely greater than required. Following which, pressure can easily be decreased to achieve desired torque value. However, the pressure cannot always easily be increased due to upper pressure limits dictated by fabrication capabilities and safety concerns.

Based on these results, the model was used to design PRA parameters for a STAR and Wedge assembly. The chosen PRA parameters are shown in Table 3. In view of proposed design considerations from minimum PRA angle was chosen to allow for 15° of hip extension during walking. Wedge PRA parameters were chosen based on STAR PRA parameters; since Wedges should retain their angular setting and not be compressed, it is best that the Wedge PRA angle be less than or equal to the minimum STAR PRA angle.

**TABLE 3:** PRA parameters.

STAR PRA	Wedge PRA
L = 138 mm	L = 138 mm
w = 100 mm	w = 100 mm
Aspect Ratio L/w = 1.38	Aspect Ratio L/w = 1.38
*n* = 5	*n* = 2 per side
min *φ* = 19°	φ = 18°
r_0_ = 17 mm	

### 3.2 Performance Testing

#### 3.2.1 Torque Test

**FIGURE 7:**
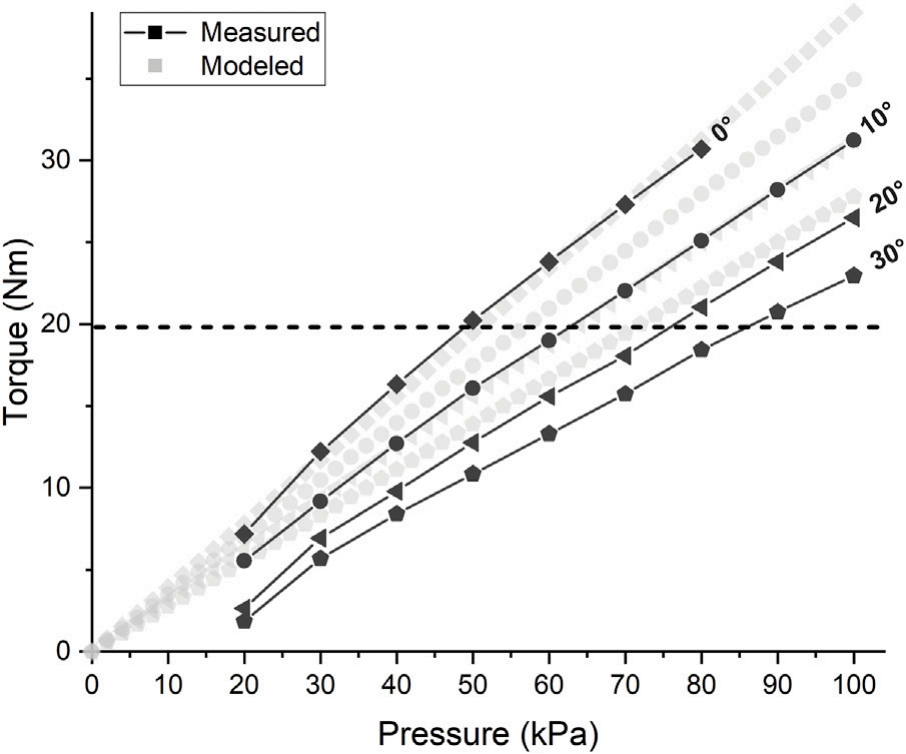
Torque Test Results: Torque vs. Pressure of SWAG actuation system. Horizontal dashed line indicates target torque output of 19.8 Nm.

Figure 7 shows the torque output at varying device angles (0–30°) and pressures (20–100 kPa). The measured torque output decreases with increased angular position similar to the model-precited values. However, the measured torque generated by the actuation system is lower than model-projected values. This is likely due to losses resulting from small deflections at the attachment points to the torque measurement apparatus and in the soft inflatable beams (TJBs). This theory is supported by the increase in deviation from predicted values as pressure increases. Higher pressure causes greater deflection, leading to larger PRA angles than intended and, consequently, a measured torque further below the predicted value. Even so, this condition may more accurately represent the true use condition since the human body surface is easily compressible and will lead to deflection at the device attachment points.

The actuation system is successfully able to supply the target torque output of 19.8 Nm (indicated by the horizontal dashed line in Figure 7) at the nominal target position of 30°. This torque is achieved at a pressure of approximately 86 kPa.

#### 3.2.2 Dynamic Pressure Test

**FIGURE 8:**
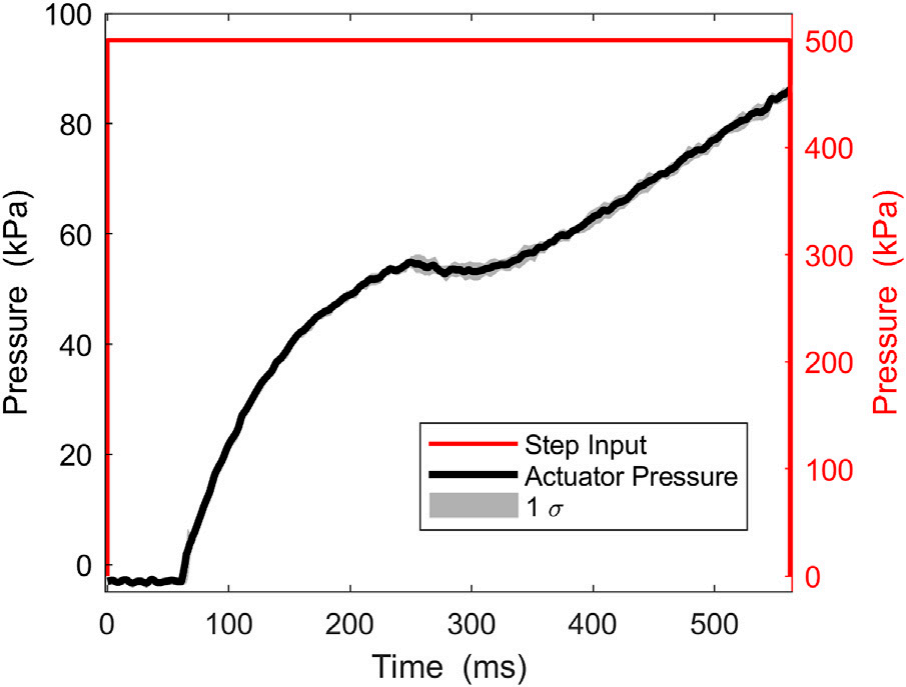
Dynamic pressure test results.

Figure 8 shows the step pressure input of 500 kPa and the resulting actuator pressure response. A delay of about 60 ms is seen before pressurization significantly starts. This is likely when air is pressurizing the network of tubing and initially filling the actuators which are rapidly increasing in volume. Next, a nonlinear response can be seen due to the interaction between the pressurization and continued volumetric expansion of the actuators. The pressure trajectory gradually becomes linear as the volumetric expansion slows down. Pressurization increases until the target pressure 86kPa, which occurs at an average of 0.56 s.

#### 3.2.3 Human Subject Test

**FIGURE 9:**
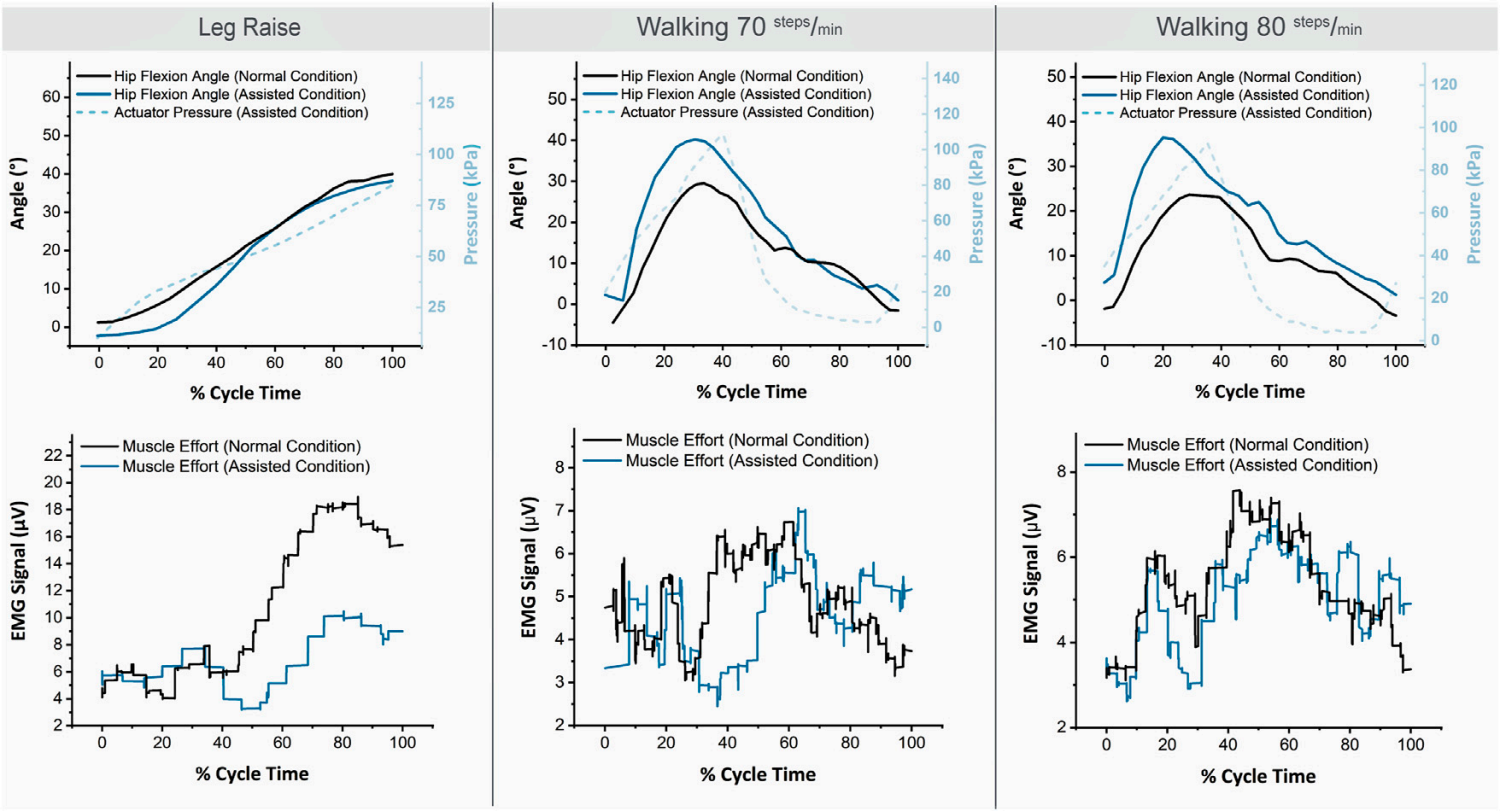
Example IMU, pressure, and EMG trajectories for one subject (S01) for the three motions and two conditions.

Figure 9 shows example IMU, pressure, and EMG results for one subject (S01). The angle trajectories for the assisted condition appear similar, although slightly higher, than those of the normal condition. The actuator pressure trajectory shown indicates the soft actuator pressurization level relative to time in the motion cycle for the assisted condition. It roughly coincides with the hip flexion angle trajectory, as is expected. For the walking conditions, actuator pressure displays a peak and rapidly declines as the actuator deflates during hip extension.

For the Leg Raise motion, the EMG values are notably lower for the assisted condition. For Walking motion, the decrease in EMG values for the assisted condition is less prominent but still observable.

**FIGURE 10:**
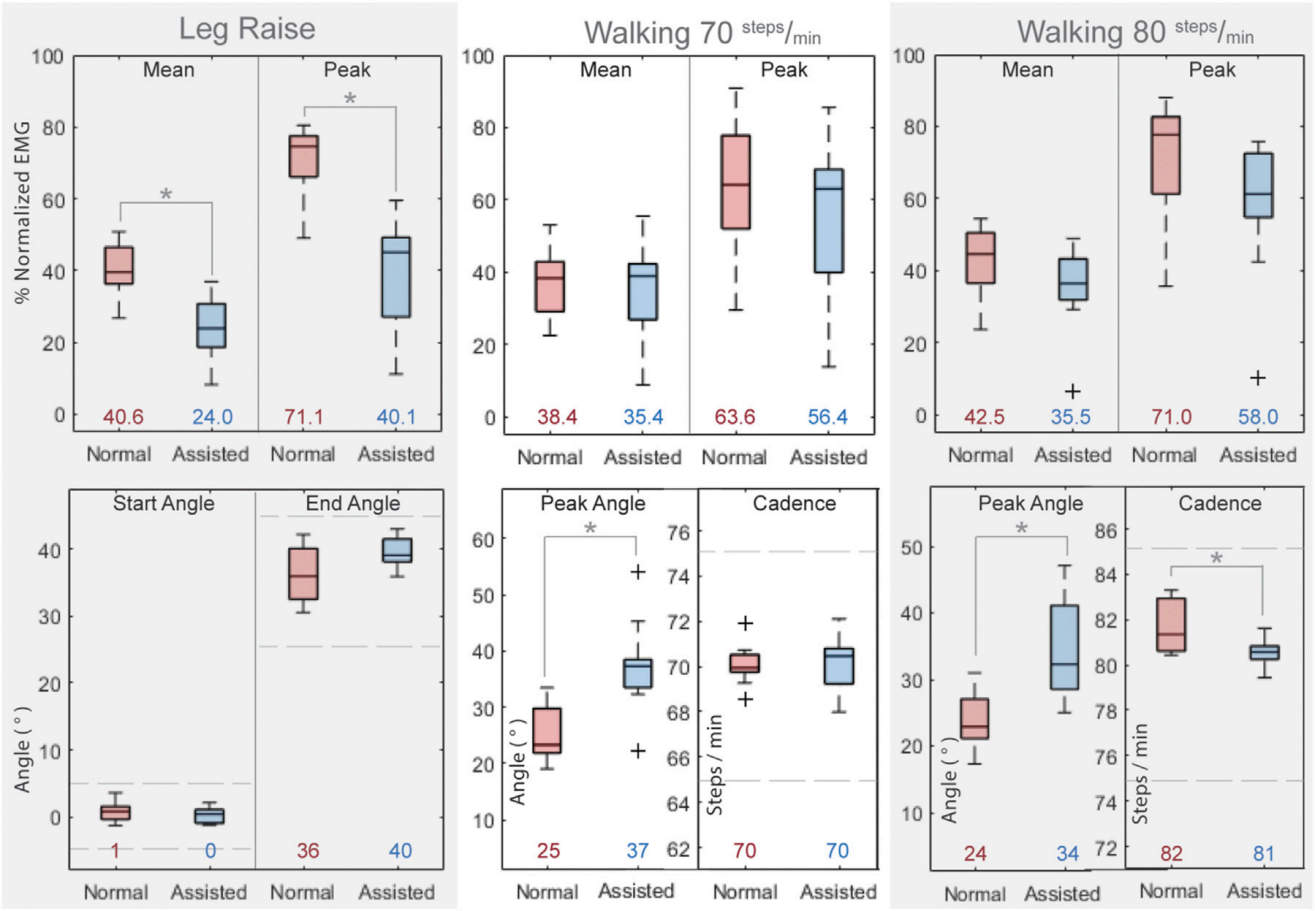
Results of Human Subject Testing. Top Row: Normalized rectus femoris muscle activation values for all subjects for the three motions and two conditions. Bottom Row: Kinematic attributes for three motions and two conditions. Asterisks indicate statistical significance. Horizontal bars in boxes show the median value and colored numbers below boxes show the mean value for each respective configuration. Dashed lines approximate kinematic limits described in section 2.3.1. Data Analysis.

Figure 10 shows the resulting normalized EMG values for the three motions and two conditions assessed. For all three motions, and for both mean muscle activation and peak muscle activation, the averaged muscle effort was lower in the assisted condition. The difference in muscle effort is minimal for Walking at 70 steps/min, slightly more prominent for Walking at 80 steps/min, and most pronounced for the Leg Raise motion. For Leg Raise, a 40.9% decrease for mean normalized EMG, and a 43.5% decrease for maximum normalized EMG are observed, both of which are statistically significant results (*p*=<0.001 and *p*=<0.001, respectively). For Walking at 70 steps/min, a 7.6% decrease for mean normalized EMG, and an 11.3% decrease for maximum normalized EMG are observed, although statistical significance was not found (*p* = 0.54 and *p* = 0.49, respectively). For Walking at 80 steps/min, a 16.4% decrease for mean normalized EMG, and an 18.3% decrease for maximum normalized EMG are observed, although statistical significance was not found (*p* = 0.08 and *p* = 0.16, respectively). While statistical significance was not found for Walking results, an observable trend of decrease in muscle activation for the assisted condition is noted.

Kinematic attributes of the trials assessed are also shown for the three motions and two conditions. Recall from section 2.3.1. Data Analysis that Leg Raise motion cycles with a peak angle of 35 ± 10° and a starting angle of 0 ± 5° were assessed, and Walking motion cycles within 5 steps/min of the respective target cadence (70 steps/min or 80 steps/min) were assessed.

The hip angle trajectories are similar for baseline and assisted conditions for Leg Raise motion in the starting angle and peak angle (*p* = 0.21 and *p* = 0.08, respectively). The starting and peak hip angles are similar for baseline and assisted conditions for Walking motion but tend to be higher for the assisted condition (Walking 70 steps/min: *p* = 0.002 and *p* = 0.01, respectively; Walking 80 steps/min: *p* = 0.007 and *p* = 0.01, respectively). The cadence is similar for both normal and assisted conditions for Walking motion (Walking 70 steps/min: *p* = 0.81, Walking 80 steps/min: *p* = 0.02).

#### 3.2.4 Dynamic Torque Assessment

**FIGURE 11:**
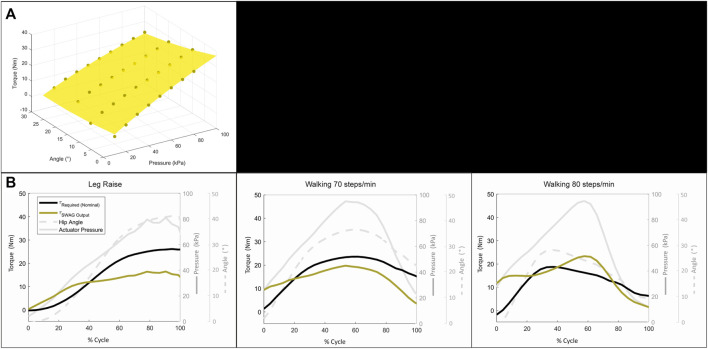
Dynamic torque assessment: **(A)** Surface fit for estimation of device torque output. **(B)** Results of dynamic torque assessment.

Figure 11A shows the second order surface fit described by Eq. 14 used to estimate the device torque output based on the results of the torque test. Figure 11B shows the estimated torque required and estimated device torque output for the three motions. The trajectory of the supplied torque is generally similar to the required torque, although it could be improved in some aspects. All three motions show an oversupply of torque at the start of the motion. This may be due to the large supply pressure and large airflow rate which are needed to achieve a sufficient torque output within the required cycle speed. For Leg Raise, the torque output is lower than the required torque starting at around 40% of cycle motion. However, as reported in Figure 10, the device is still able to provide significant assistance for the Leg Raise motion. For Walking at 70 steps/min, the device output torque follows the required torque trajectory fairly well from around 20–60% of cycle motion. Torque output decreases more rapidly than torque required after 60% cycle motion, however, the device is targeted for hip flexion assistance, so hip extension is not a focus. For Walking at 80 steps/min, torque output surpasses torque required from about 40 to 80% of cycle motion. This occurs during hip extension, where actuator angle is decreasing, which causes an increase in pressure. During hip extension, actuator pressure should be released. Comparing the angle and torque trajectories (grey dashed line and grey solid line, respectively), the pressure peaks after hip angle peaks. This suggests the device and user synchronization can be improved to better assistance efficacy.

## 4 Discussion

### 4.1 Discussion of Results

The increased peak hip angle observed for the assisted walking conditions may be due, in part, to IMU sensor shifting. It may also be due to subjects adjusting their walking pattern to the position of the device. This could plausibly be mitigated by tuning the actuation angle and timing of the device to better suit a subject’s gait pattern and improving positioning of the device and sensors.

The results of the torque test show the actuation system of the device to be capable of the magnitude of torque required for hip flexion assistance. Based on this result, the device performed as expected when applied to the Leg Raise motion. The device shows a clear reduction in muscle effort, which demonstrates that it is able to successfully provide hip flexion assistance to a subject.

In the Walking motion, however, the device does not yet show comparable results with some electric-motor-driven solutions (Panizzolo et al., 2016; Quinlivan et al., 2017; Wu et al., 2020; Panizzolo et al., 2021; Tricomi et al., 2022; Asbeck et al., 2015). Although the mean and maximum muscle efforts are reduced on average, this is not the case for each subject individually, and statistical significance is not found. Some subjects showed clear benefit while others showed minimal change and still others showed notable hindrance with suit assistance. The long whiskers and largely overlapping boxes of the walking box plots in Figure 10 are the product of these mixed results for walking assistance. The suit produces clearly positive results for Leg Raise motion and should theoretically be able to provide assistance for flexion during walking as well. In this work, a simple control is used where the device is set to a speed and the user must adjust his or her pace to that speed. Since walking requires more coordination with the device than leg raising, the discrepancy in results between these motions may arise from poor device-subject coordination. It is reasonably expected that incorporating a user intent detection control mechanism can improve walking assistance efficacy levels, as such control mechanisms have already been successful for walking assistive devices with the same type of actuators (PRAs) applied at the knee joint (Sridar et al., 2020, 2017).

Increasing device speed may also help coordination. This hypothesis is supported in that assistance results are observably better for the faster walking speed of 80 steps/min. Several subjects commented that they found the cadence of 80 steps/min more comfortable. A moderate human walking cadence is reported to be around 100 steps/min (Winter 1984; Tudor-Locke et al., 2019), so it follows that subjects would find 80 steps/min more natural and plausibly find better coordination with the device at this cadence. The control strategy and bandwidth are not optimized in this work but could be explored in future work. By implementing a more complex control strategy, coordination could be improved, and by adjusting input pressure and set point pressure bandwidth could be improved to increase device speed. Additionally, future work could include a study wherein subjects are asked to more extensively practice moving while assisted by the device before efficacy data is recorded, which would likely improve device-subject coordination. A longitudinal study could also be performed to assess assistance efficacy over use time, as likely the subject would become increasingly more comfortable using the device and coordinating to optimize assistance.

**FIGURE 12:**
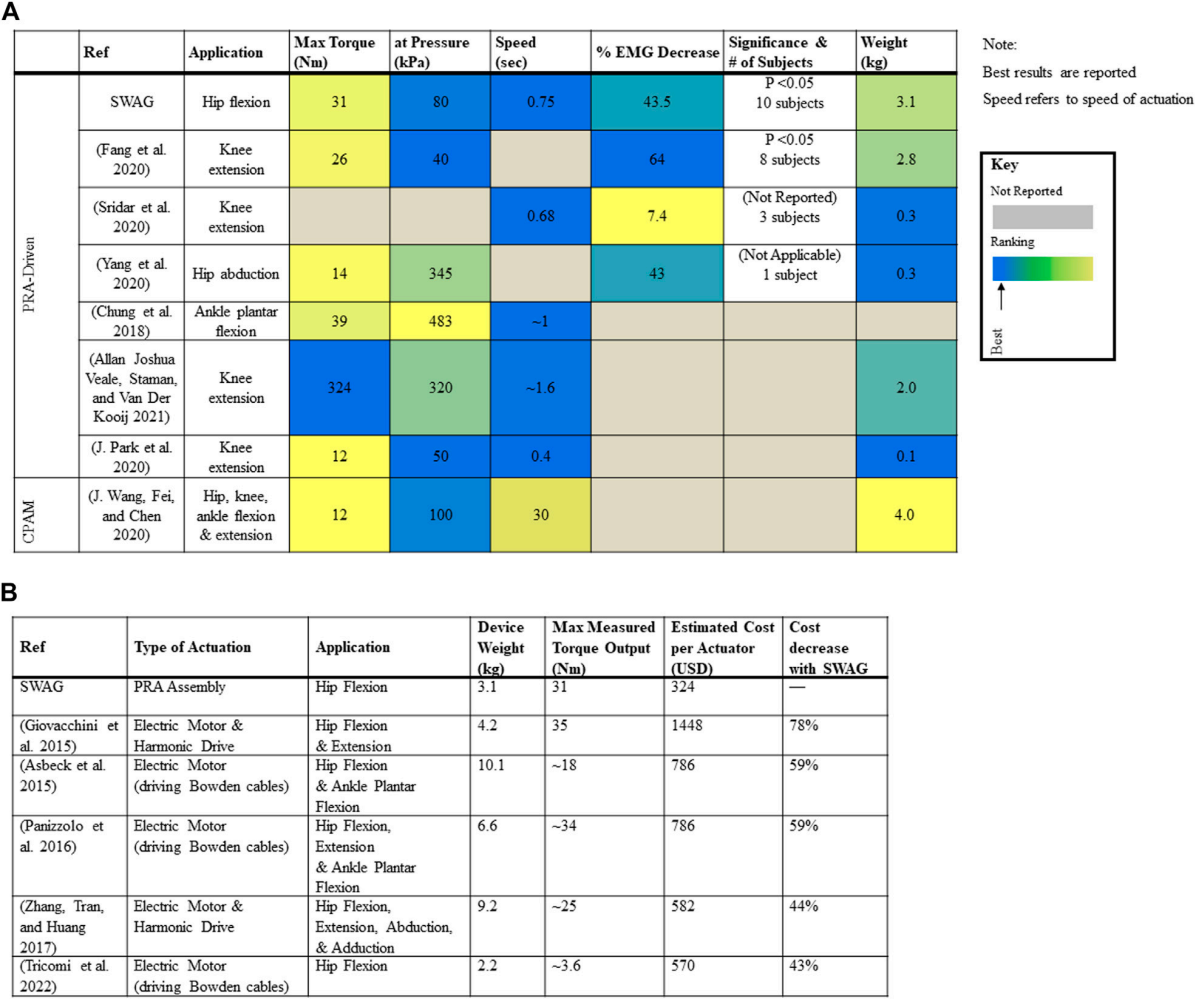
Comparison of SWAG with other devices: **(A)** Comparison with Contemporary Lower Limb Devices Driven by Soft Bending Actuators. **(B)** Comparison with Contemporary Hip Devices Driven by Electric Motor Actuation.

Figure 12A shows a comparison of the capabilities of the SWAG device with other state-of-the-art lower limb assistive devices driven by soft bending actuators. The SWAG device is commensurate with contemporaries for torque output and requires a comparatively low supply pressure. The device performs reasonably well in terms of speed, despite the relatively large volume of its actuation system, and the weight of the device is in range with its peers.

Figure 12B shows a comparison of the weight, torque capabilities, and cost of the SWAG device with hip exoskeletons driven by electric motor actuation, including some state of the art traditional systems (Kapsalyamov et al., 2019) as well as some state of the art cable-driven soft systems. The cost of the SWAG actuator is 43% cheaper than that presented by Tricomi et al., 44% cheaper than that presented by Zhang et al., 59% cheaper than those presented by Asbeck et al. and Panizzolo et al., and 78% cheaper than that presented by Giovacchini et al. (Giovacchini et al., 2015; Panizzolo et al., 2016; Ting Zhang et al., 2017; Asbeck et al., 2015; Tricomi et al., 2022). The SWAG device weight and torque output are in range with contemporary electromechanically powered hip exoskeletons. While there exist successful actuation systems lower in cost ((Seo, Lee, and Park 2017) estimated 116 USD), there also exist many higher in cost ((Juanjuan Zhang et al., 2017), estimated 3020 USD), and the SWAG actuation assembly is generally notably lower in cost. Furthermore, its fabrication materials are accessible and fabrication methods are simple. The goal of this work is to develop a more accessible device. The main advantage presented by SWAG to the state of the art is its cost and accessibility.

The SWAG device can also be complementary with existing rigid, electromechanically actuated systems in that such systems are ideal for patients suffering from a high level of impairment who need professional attention in a rehabilitation facility and perhaps cannot support their own bodyweight. Gradually, as such a patient improves, he or she could begin to use a device like SWAG at home for rehabilitation. Before SWAG is introduced, the orthopedic characteristics of the patient should be assessed to determine compatibility with SWAG. For example joint stiffness, joint torque, and range of motion could be assessed as in (Chaparro-Rico et al., 2020).

Only a few groups have assessed their device with human subjects. Several groups have measured actuator performance metrics, such as torque output, and proposed their group’s actuator is sufficient for assistance, but most have not yet undergone human testing. Of the groups that have performed human testing, many assessed other attributes such as suit range of motion and sensing capabilities, but they have yet to assess assistance efficacy. A small number of groups performed pilot tests with one subject, but very few assessed the efficacy of device assistance across a group of subjects. Therefore, the SWAG device is one of the first of its kind to be developed to the point of assessment with humans and, furthermore, assessed for assistance efficacy.

### 4.2 Limitations

Study limitations: The upper IMU would ideally be placed at the pelvis but was placed at the torso of the subject for practical reasons. This may give a less accurate measure of hip flexion compared to a measurement anchored at the pelvis. Since this setting was consistent across both conditions, and results depend upon comparison between conditions, this setting is presumed to have an insignificant impact on results. Additionally, the suit attachments are maximally large to distribute force, but this also means they will almost inevitably cover some body areas where the IMU and EMG sensors must be attached and, thus, contact these sensors, which could lead to noise or shifting. By testing a substantial number of subjects, and since subjects vary in size such that suit and sensors fall in different positions relative to one another across subjects, it is presumed that the mean results are not significantly affected.

Device limitations. Although actuation speed is proportionate to similar devices, it is not yet fast enough to achieve the moderate walking pace of 100 steps/min, but is currently able to support approximately 80 steps/min. Due to the mentioned large volume of the STAR PRAs, the speed of the device actuation is largely restricted by the volumetric flowrate of the air, including both inflation and deflation. Future development work might include a combination of decreasing actuator volume (as demonstrated by (Sridar et al., 2020)) and increasing volumetric flow rate, to increase the available walking speed which can be assisted by SWAG. Future work may also include additional joints (knee, ankle) as well as additional actuation directions (hip extension).

## 5 Conclusion

We have presented SWAG, a PRA-driven exoskeleton for hip flexion rehabilitation, which is made from lightweight, flexible, and low-cost materials. Through mechanical testing, we have demonstrated the torque output capabilities of the actuation system to be of sufficient magnitude for hip flexion assistance applications. We have subsequently shown an assessment of the overall device through human subject testing and found the device effective to assist in hip flexion for Leg Raise motion and bearing potential to assist walking motion. Finally, we have compared the device with other soft-bending-actuator-driven lower limb assistive devices and shown SWAG to be among the first of its kind and feasibly the furthest developed device for hip flexion assistance. Based on the positive results presented in this work, the SWAG device has potential to be further developed to afford a more accessible rehabilitation option to those who suffer from lower limb immobility.

## Data Availability

The raw data supporting the conclusions of this article will be made available by the authors, without undue reservation.

## References

[B1] Asbeck A. T. De Rossi S. M. M. Holt K. G. Walsh C. J. (2015a). A Biologically Inspired Soft Exosuit for Walking Assistance. Int. J. Robotics Res. 34, 744–762. 10.1177/0278364914562476

[B2] Asbeck A. T. Dyer R. J. Larusson A. F. Walsh C. J. (2013). “Biologically-Inspired Soft Exosuit,” in 2013 IEEE 13th International Conference on Rehabilitation Robotics (ICORR), Seattle, WA, June 24–26, 2013, 1–8. 10.1109/ICORR.2013.6650455 24187272

[B3] Asbeck A. T. Schmidt K. Walsh. C. J. (2015b). Soft Exosuit for Hip Assistance. Robotics Autonomous Syst. 73, 102–110. Elsevier. 10.1016/j.robot.2014.09.025

[B4] Awad L. N. Bae J. O’Donnell K. De Rossi S. M. M. Hendron K. Sloot L. H. (2017). A Soft Robotic Exosuit Improves Walking in Patients after Stroke. Sci. Transl. Med. 9, 1–12. 10.1126/scitranslmed.aai9084 28747517

[B5] Brown E. Rodenberg N. Amend J. Mozeika A. Steltz E. Zakin M. R. (2010). Universal Robotic Gripper Based on the Jamming of Granular Material. Proc. Natl. Acad. Sci. U.S.A. 107 (44), 18809–18814. 10.1073/pnas.1003250107

[B6] Carpi F. Bauer S. De Rossi D. (2010). Stretching Dielectric Elastomer Performance. Science 330 (6012), 1759–1761. Available at: http://science.sciencemag.org/content/330/6012/1759.abstract . 10.1126/science.1194773 21205659

[B7] Centers for Disease Control and Prevention (2016). Hip Fractures Among Older Adults. Available at: http://www.cdc.gov/homeandrecreationalsafety/falls/adulthipfx.html (Accessed January 26, 2022).

[B8] Centers for Disease Control and Prevention (2021). Stroke Facts.” 2021. Available at: http://www.cdc.gov/stroke/facts.htm (Accessed January 26, 2022).

[B9] Chaparro-Rico B. D. M. Cafolla D. Tortola P. Galardi G. Galardi G. (2020). Assessing Stiffness, Joint Torque and ROM for Paretic and Non-paretic Lower Limbs during the Subacute Phase of Stroke Using Lokomat Tools. Appl. Sci. 10 (18), 6168. 10.3390/APP10186168

[B10] Chou C-P. Hannaford B. (1996). Measurement and Modeling of McKibben Pneumatic Artificial Muscles. IEEE Trans. Robot. Automat. 12 (1), 90–102. 10.1109/70.481753

[B11] Chung J. Heimgartner R. Oneill C. T. Phipps N. S. Walsh C. J. (2018). “ExoBoot, a Soft Inflatable Robotic Boot to Assist Ankle during Walking: Design, Characterization and Preliminary Tests,” in Proceedings of the IEEE RAS and EMBS International Conference on Biomedical Robotics and Biomechatronics, Enschede, Netherlands, 2018-August. 10.1109/BIOROB.2018.8487903

[B12] Cianchetti M. Ranzani T. Gerboni G. Nanayakkara T. Althoefer K. Dasgupta P. (2014). Soft Robotics Technologies to Address Shortcomings in Today's Minimally Invasive Surgery: The STIFF-FLOP Approach. Soft Robotics 1 (2), 122–131. 10.1089/soro.2014.0001

[B13] Ding Y. Galiana I. Asbeck A. Quinlivan B. De Rossi S. M. M. Walsh C. (2014). “Multi-Joint Actuation Platform for Lower Extremity Soft Exosuits,” in Proceedings - IEEE International Conference on Robotics and Automation, Hong Kong, China, May 31–June 7, 2014. 10.1109/ICRA.2014.6907024

[B14] Ding Y. Galiana I. Asbeck A. T. De Rossi S. M. M. Bae J. Santos T. R. T. (2017). Biomechanical and Physiological Evaluation of Multi-Joint Assistance with Soft Exosuits. IEEE Trans. Neural Syst. Rehabil. Eng. 25, 119–130. 10.1109/TNSRE.2016.2523250 26849868

[B15] Diteesawat R. S. Helps T. Taghavi M. Rossiter J. (2018). “High Strength Bubble Artificial Muscles for Walking Assistance,” in 2018 IEEE International Conference on Soft Robotics, RoboSoft, Livorno, Italy, April 24–28, 2018. 10.1109/ROBOSOFT.2018.8404950

[B16] Fang J. Yuan J. Wang M. Xiao L. Yang J. Lin Z. (2020). Novel Accordion-Inspired Foldable Pneumatic Actuators for Knee Assistive Devices. Soft Robotics 7 (1), 95–108. 10.1089/soro.2018.0155 31566506

[B17] Felt W. (2019). Folded-Tube Soft Pneumatic Actuators for Bending. Soft Robotics 6 (2), 174–183. 10.1089/soro.2018.0075 30912715

[B18] Giovacchini F. Vannetti F. Fantozzi M. Cempini M. Cortese M. Parri A. (2015). A Light-Weight Active Orthosis for Hip Movement Assistance. Robotics Autonomous Syst. 73, 123–134. 10.1016/j.robot.2014.08.015

[B19] Halaki M. Gi K. (2012). “Normalization of EMG Signals: To Normalize or Not to Normalize and what to Normalize to?,” in Computational Intelligence in Electromyography Analysis - A Perspective on Current Applications and Future Challenges. 10.5772/49957

[B20] Hiller J. Lipson H. (2012). Automatic Design and Manufacture of Soft Robots. IEEE Trans. Robot. 28 (2), 457–466. 10.1109/TRO.2011.2172702

[B21] Ilievski F. Mazzeo A. D. Shepherd R. F. Chen X. Whitesides G. M. (2011). Soft Robotics for Chemists. Angew. Chem. 123 (8), 1930–1935. 10.1002/ange.201006464 21328664

[B22] In H. Kang B. B. Sin M. Cho K.-J. (2015). Exo-Glove: A Wearable Robot for the Hand with a Soft Tendon Routing System. IEEE Robot. Automat. Mag. 22, 97–105. 10.1109/MRA.2014.2362863

[B23] Kapsalyamov A. Jamwal P. K. Hussain S. Ghayesh M. H. (2019). State of the Art Lower Limb Robotic Exoskeletons for Elderly Assistance. IEEE Access 7, 95075–95086. 10.1109/ACCESS.2019.2928010

[B24] Karavas N. Kim J. Galiana I. Ding Y. Couture A. Wagner D. (2017). Autonomous Soft Exosuit for Hip Extension Assistance. Biosyst. Biorob., 331–335. 10.1007/978-3-319-46532-6_54

[B25] Khin P. M. Yap H. K. Ang M. H. Yeow C.-H. (2017). “Fabric-based Actuator Modules for Building Soft Pneumatic Structures with High Payload-To-Weight Ratio,” in 2017 IEEE/RSJ International Conference on Intelligent Robots and Systems (IROS), vancouver, BC, Canada, September 24–28, 2017, 2744–2750. 10.1109/IROS.2017.8206102

[B26] Kim S. Laschi C. Trimmer B. (2013). Soft Robotics: A Bioinspired Evolution in Robotics. Trends Biotechnol. 31 (5), 287–294. 10.1016/j.tibtech.2013.03.002 23582470

[B27] Kocis P. Knoflicek R. (2017). Artificial Muscles: State of the Art and a New Technology. Mm Sj 2017, 1668–1673. 10.17973/MMSJ.2017_02_2016100

[B28] Krishnan C. Kotsapouikis D. Dhaher Y. Y. Rymer W. Z. (2013). Reducing Robotic Guidance during Robot-Assisted Gait Training Improves Gait Function: A Case Report on a Stroke Survivor. Arch. Phys. Med. Rehabil. 94, 1202–1206. 10.1016/j.apmr.2012.11.016 23168401

[B29] Kwon S. H. Lee B. S. Lee H. J. Kim E. J. LeeLee J. A. YangYang S. P. (2020). Energy Efficiency and Patient Satisfaction of Gait with Knee-Ankle-Foot Orthosis and Robot (Rewalk)-Assisted Gait in Patients with Spinal Cord Injury. Ann. Rehabil. Med. 44 (2), 131–141. 10.5535/arm.2020.44.2.131 32392652 PMC7214138

[B30] Li Y. Tian M. Wang X. (2019). “Fuzzy Self-Tuning PID Control of a Lower Limb Soft Exosuit Based on Pneumatic Artificial Muscles,” in Proceedings of 2019 IEEE 3rd Advanced Information Management, Communicates, Electronic and Automation Control Conference, IMCEC, Chongqing, China, October 11–13, 2019. 10.1109/IMCEC46724.2019.8983956

[B31] Low F.-Z. Tan H. H. Lim J. H. Yeow C.-H. (2016). Development of a Soft Pneumatic Sock for Robot-Assisted Ankle Exercise. J. Med. Devices 10 (1), 14503–14505. 10.1115/1.4032616

[B32] Low J.-H. Delgado-Martinez I. Yeow C.-H. (2014). Customizable Soft Pneumatic Chamber-Gripper Devices for Delicate Surgical Manipulation. J. Med. Devices 8 (4), 44504–44505. 10.1115/1.4027688

[B33] Low J. H. Lee W. W. Khin P. M. Thakor N. V. Kukreja S. L. Ren H. L. (2017). Hybrid Tele-Manipulation System Using a Sensorized 3-D-Printed Soft Robotic Gripper and a Soft Fabric-Based Haptic Glove. IEEE Robot. Autom. Lett. 2 (2), 880–887. 10.1109/LRA.2017.2655559

[B34] Meng J. Gerez L. Chapman J. Liarokapis M. (2020). “A Tendon-Driven, Preloaded, Pneumatically Actuated, Soft Robotic Gripper with a Telescopic Palm,” in 2020 3rd IEEE International Conference on Soft Robotics, RoboSoft, New Haven, CT, May 15–July 15, 2020. 10.1109/RoboSoft48309.2020.9115986

[B35] Miller-Jackson T. M. Li J. Natividad R. F. Yeow R. C.-H. (2019). “STAS: An Antagonistic Soft Pneumatic Actuator Assembly for High Torque Output,” in RoboSoft 2019 - 2019 IEEE International Conference on Soft Robotics, Seoul, Korea, April 14–18, 2019. 10.1109/ROBOSOFT.2019.8722791

[B36] Miller-Jackson T. Sun Y. Natividad R. Yeow C. H. (2019). Tubular Jamming: A Variable Stiffening Method toward High-Force Applications with Soft Robotic Components. Soft Rob. 6, 468–482. 10.1089/soro.2018.0084 31158061

[B37] Natividad R. F. Miller-Jackson T. Chen-Hua R. Y. (2020). A 2-DOF Shoulder Exosuit Driven by Modular, Pneumatic, Fabric Actuators. IEEE Trans. Med. Robot. Bionics 3 (1), 166–178. 10.1109/tmrb.2020.3044115

[B38] Panizzolo F. A. Annese E. Paoli A. Marcolin G. (2021). A Single Assistive Profile Applied by a Passive Hip Flexion Device Can Reduce the Energy Cost of Walking in Older Adults. Appl. Sci. 11 (6), 2851. 10.3390/app11062851

[B39] Panizzolo F. A. Galiana I. Asbeck A. T. Siviy C. Schmidt K. Holt K. G. (2016). A Biologically-Inspired Multi-Joint Soft Exosuit that Can Reduce the Energy Cost of Loaded Walking. J. Neuroeng. Rehabil. 13 (1). 10.1186/s12984-016-0150-9 PMC4864923 27169361

[B40] Park J. Choi J. Kim S. J. Seo K.-H. Kim J. (2020). Design of an Inflatable Wrinkle Actuator with Fast Inflation/Deflation Responses for Wearable Suits. IEEE Robot. Autom. Lett. 5 (3), 3799–3805. 10.1109/LRA.2020.2976299

[B41] Park Y.-L. Chen B.-r. Pérez-Arancibia N. O. Young D. Stirling L. Wood R. J. (2014a). Design and Control of a Bio-Inspired Soft Wearable Robotic Device for Ankle-Foot Rehabilitation. Bioinspir. Biomim. 9, 016007. 10.1088/1748-3182/9/1/016007 24434598

[B42] Park Y.-L. Santos J. Galloway K. G. Goldfield E. C. Wood R. J. (2014b). “A Soft Wearable Robotic Device for Active Knee Motions Using Flat Pneumatic Artificial Muscles,” in Proceedings - IEEE International Conference on Robotics and Automation, Hong Kong, China, May 31–June 7, 2014. 10.1109/ICRA.2014.6907562

[B43] Parvataneni K. Ploeg L. Olney S. J. Brouwer B. (2009). Kinematic, Kinetic and Metabolic Parameters of Treadmill versus Overground Walking in Healthy Older Adults. Clin. Biomech. 24 (1), 95–100. 10.1016/j.clinbiomech.2008.07.002 18976839

[B44] Quinlivan B. T. Lee S. Malcolm P. Rossi D. M. Grimmer M. Siviy C. (2017). Assistance Magnitude versus Metabolic Cost Reductions for a Tethered Multiarticular Soft Exosuit. Sci. Robot. 2 (2), eaah4416. 10.1126/scirobotics.aah4416 33157865

[B45] Ramakrishnan H. K. Kadaba M. P. (1991). On the Estimation of Joint Kinematics during Gait. J. Biomech. 24 (10), 969–977. 10.1016/0021-9290(91)90175-M 1744154

[B46] Rus D. Tolley M. T. (2015). Design, Fabrication and Control of Soft Robots. Nature 521, 467–475. 10.1038/nature14543 26017446

[B47] Sawicki G. S. Ferris D. P. (2009). A Pneumatically Powered Knee-Ankle-Foot Orthosis (KAFO) with Myoelectric Activation and Inhibition. J. Neuroengineering Rehabil. 6 (1), 23. 10.1186/1743-0003-6-23 PMC2717982 19549338

[B48] Sczesny-Kaiser M. Trost R. Aach M. Schildhauer T. A. Schwenkreis P. Tegenthoff M. (2019). A Randomized and Controlled Crossover Study Investigating the Improvement of Walking and Posture Functions in Chronic Stroke Patients Using HAL Exoskeleton - the HALESTRO Study (HAL-Exoskeleton STROke Study). Front. Neurosci. 13, 1–13. 10.3389/fnins.2019.00259 30983953 PMC6450263

[B49] Seo K. Lee J. Park Y. J. (2017). “Autonomous Hip Exoskeleton Saves Metabolic Cost of Walking Uphill,” in IEEE International Conference on Rehabilitation Robotics, London, United Kingdom, July 17–20, 2017. 10.1109/ICORR.2017.8009254 28813826

[B50] Sridar S. Nguyen P. H. Zhu M. Lam Q. P. Polygerinos P. (2017). “Development of a Soft-Inflatable Exosuit for Knee Rehabilitation,” in IEEE International Conference on Intelligent Robots and Systems, Vancouver, BC, Canada, 2017-September. 10.1109/IROS.2017.8206220

[B51] Sridar S. Poddar S. Tong Y. Polygerinos P. Zhang W. (2020). Towards Untethered Soft Pneumatic Exosuits Using Low-Volume Inflatable Actuator Composites and a Portable Pneumatic Source. IEEE Robot. Autom. Lett. 5 (3), 4062–4069. 10.1109/LRA.2020.2986744

[B52] Sun H. Chen X.-P. (2014). Towards Honeycomb PneuNets Robots. Editors Kim J-H Matson E. T Myung H Xu P Karray F (Cham: Springer International Publishing). 10.1007/978-3-319-05582-4_28

[B53] Sun Y. Yap H. K. Liang X. Guo J. Qi P. Ang M. H. (2017). Stiffness Customization and Patterning for Property Modulation of Silicone-Based Soft Pneumatic Actuators. Soft Rob. 4 (3), 251–260. 10.1089/soro.2016.0047 29182082

[B54] The World Bank (2022). Current Health Expenditure Per Capita (Current US$). Available at: https://data.worldbank.org/indicator/SH.XPD.CHEX.PC.CD?end=2019&name_desc=false&start=2000&view=chart .

[B55] Tricomi E. Lotti N. Missiroli F. Zhang X. Xiloyannis M. Muller T. (2022). Underactuated Soft Hip Exosuit Based on Adaptive Oscillators to Assist Human Locomotion. IEEE Robot. Autom. Lett. 7 (2), 936–943. 10.1109/LRA.2021.3136240

[B56] Tudor-Locke C. Aguiar E. J. Han H. Ducharme S. W. Schuna J. M. Barreira T. V. (2019). Walking Cadence (Steps/Min) and Intensity in 21-40 Year Olds: CADENCE-Adults. Int. J. Behav. Nutr. Phys. Act 16 (1), 8. 10.1186/s12966-019-0769-6 30654810 PMC6337834

[B57] Veale A. J. Staman K. van der Kooij H. (2019). Realizing Soft High Torque Actuators for Complete Assistance Wearable Robots. Biosyst. Biorob. 22, 39–43. 10.1007/978-3-030-01887-0_8

[B58] Veale A. J. Staman K. Van Der Kooij H. (2021). Soft, Wearable, and Pleated Pneumatic Interference Actuator Provides Knee Extension Torque for Sit-To-Stand. Soft Rob. 8 (1), 28–43. 10.1089/soro.2019.0076 32364831

[B59] Veneman J. F. Kruidhof R. Hekman E. E. G. Ekkelenkamp R. Van Asseldonk E. H. F. van der Kooij H. (2007). Design and Evaluation of the LOPES Exoskeleton Robot for Interactive Gait Rehabilitation. IEEE Trans. Neural Syst. Rehabil. Eng. 15 (3), 379–386. 10.1109/TNSRE.2007.903919 17894270

[B60] Walpole S. C. Prieto-Merino D. Edwards P. Cleland J. Stevens G. Roberts I. (2012). The Weight of Nations: An Estimation of Adult Human Biomass. BMC Public Health 12, 439. 10.1186/1471-2458-12-439 22709383 PMC3408371

[B61] Wang J. Fei Y. Chen W. (2020). Integration, Sensing, and Control of a Modular Soft-Rigid Pneumatic Lower Limb Exoskeleton. Soft Rob. 7 (2), 140–154. 10.1089/soro.2019.0023 31603736

[B62] Wang Z. Chen M. Z. Q. Yi J. (2015). Soft Robotics for Engineers. HKIE Trans. 22 (2), 88–97. 10.1080/1023697X.2015.1038321

[B63] Whiteley R. Hansen C. Thomson A. Sideris V. Wilson M. G. (2021). Lower Limb EMG Activation during Reduced Gravity Running on an Incline. Speed Matters More Than Hills Irrespective of Indicated Bodyweight. Gait Posture 83, 52–59. 10.1016/j.gaitpost.2020.09.029 33075719

[B64] Winter D. A. (1984). Kinematic and Kinetic Patterns in Human Gait: Variability and Compensating Effects. Hum. Move. Sci. 3, 51, 76. 10.1016/0167-9457(84)90005-8

[B65] Winter D. A. (2009). Biomechanics and Motor Control of Human Movement. Fourth Edition. Waterloo, ON, Canada: Wiley. 10.1002/9780470549148

[B66] Wu X. Fang K. Chen C. Zhang Y. (2020). Development of a Lower Limb Multi-Joint Assistance Soft Exosuit. Sci. China Inf. Sci. 63, 170207. 10.1007/s11432-019-2812-7

[B67] Xiloyannis M. Alicea R. Georgarakis A.-M. Haufe F. L. Wolf P. Masia L. (2021). Soft Robotic Suits: State of the Art, Core Technologies, and Open Challenges. IEEE Trans. Robot., 1–20. 10.1109/tro.2021.3084466

[B68] Yang H. D. Cooper M. Akbas T. Schumm L. Orzel D. Walsh C. J. (2020). “A Soft Inflatable Wearable Robot for Hip Abductor Assistance: Design and Preliminary Assessment,” in Proceedings of the IEEE RAS and EMBS International Conference on Biomedical Robotics and Biomechatronics, 2020-November, New York, NY. 10.1109/BioRob49111.2020.9224283

[B69] Yap H. K. Goh J. C. H. Yeow R. C. H. (2015). “Design and Characterization of Soft Actuator for Hand Rehabilitation Application,” in 6th European Conference of the International Federation for Medical and Biological Engineering: MBEC 2014, 7-11 September 2014, Dubrovnik, Croatia. Editors Lacković I. Vasic D.(Cham: Springer International Publishing), 367, 370 . 10.1007/978-3-319-11128-5_92

[B70] Yap H. K. NgNg H. Y. Yeow C.-H. (2016). High-Force Soft Printable Pneumatics for Soft Robotic Applications. Soft Robotics 3 (3), 144–158. 10.1089/soro.2016.0030

[B71] Zhang J. Fiers P. Witte K. A. Jackson R. W. Poggensee K. L. Atkeson C. G. (2017). Human-in-the-Loop Optimization of Exoskeleton Assistance during Walking. Science 356 (6344), 1280–1284. 10.1126/science.aal5054 28642437

[B72] Zhang T. Tran M. HuangHuang H. H. (2017). “NREL-exo: A 4-DoFs Wearable Hip Exoskeleton for Walking and Balance Assistance in Locomotion,” in IEEE International Conference on Intelligent Robots and Systems, Vancouver, BC, Canada, 2017-September. 10.1109/IROS.2017.8202201

